# Chemical proteomics reveals sinomenine’s anti-inflammatory mechanism through serum protein covalent modification

**DOI:** 10.1186/s13020-026-01403-2

**Published:** 2026-04-30

**Authors:** Meixian Liu, Zhiyuan Zheng, Yida Zhang, Xiqing Bian, Yue Zhuo, Hai-Ying Wu, Jian-Lin Wu, Na Li

**Affiliations:** 1 State Key Laboratory of Mechanism and Quality of Chinese Medicine, Macau University of Science and Technology, Avenida Wai Long, Taipa, 999078 Macau China; 2 Translational Innovation Center, Shenzhen Bay Laboratory, Shenzhen, 518132 China; 3 Guangdong Provincial Key Laboratory of Cell and Gene Therapy, Faculty of Pharmaceutical Sciences, Shenzhen University of Advanced Technology, Shenzhen, 518107 China; 4 College of Medical Technology and Engineering, Henan University of Science and Technology, Luoyang, 471023 China; 5 School of Pharmacy, Macau University of Science and Technology, Taipa, Macao SAR China; 6 Science and Technology Innovation Center, Guangzhou University of Chinese Medicine, Guangzhou, 510405 China; 7 Emergency Department, First Affiliated Hospital of Kunming Medical University, Kunming, 650032 China

**Keywords:** Sinomenine, Covalent protein modifications, Serum proteomics, Anti-inflammatory effect, Kallikrein-kinin system, Coagulation system, Complement system

## Abstract

**Background:**

Covalent protein modification by drugs or their reactive metabolites has emerged as an important mechanism underlying pharmacological activity. However, its contribution to the therapeutic effects of compounds derived from traditional Chinese medicine remains insufficiently characterized. Sinomenine (SIN), an active alkaloid from *Sinomenium acutum*, has long been utilized in managing inflammatory disorders, yet the molecular basis of its efficacy is not fully elucidated.

**Methods:**

A discovery-driven chemical proteomics approach was deployed to investigate the role of covalent protein modification in the action of SIN. Metabolite profiling and in vitro reactivity assays were initially conducted to evaluate the modification potential of SIN and its metabolites. Subsequently, in vivo serum proteomics analysis in rat was performed to map the covalent modification landscape. Enriched biological pathways were identified through bioinformatics analyses, and functional validation was executed using western blotting and enzymatic activity assays.

**Results:**

Results demonstrated that SIN, along with its oxygenated and demethylated metabolites, could covalently modify cysteine and lysine residues on proteins via three distinct modification patterns. Serum proteomics analysis identified seven proteins modified by SIN in vivo. Pathway enrichment analysis revealed that these target proteins were predominantly involved in coagulation and complement pathways. Functional validation demonstrated that SIN treatment significantly suppressed the activation of the coagulation cascade, the kallikrein-kinin system (KKS), and the complement cascade.

**Conclusions:**

This study provides evidence that covalent protein modification may contribute to the pharmacological effects of SIN by modulating coagulation and complement pathways, which are implicated in inflammatory regulation. The findings offer new mechanistic insights into SIN’s action and underscore the utility of covalent proteomics as an effective strategy for uncovering the molecular mechanisms of bioactive compounds derived from traditional Chinese medicine.

**Graphical Abstract:**

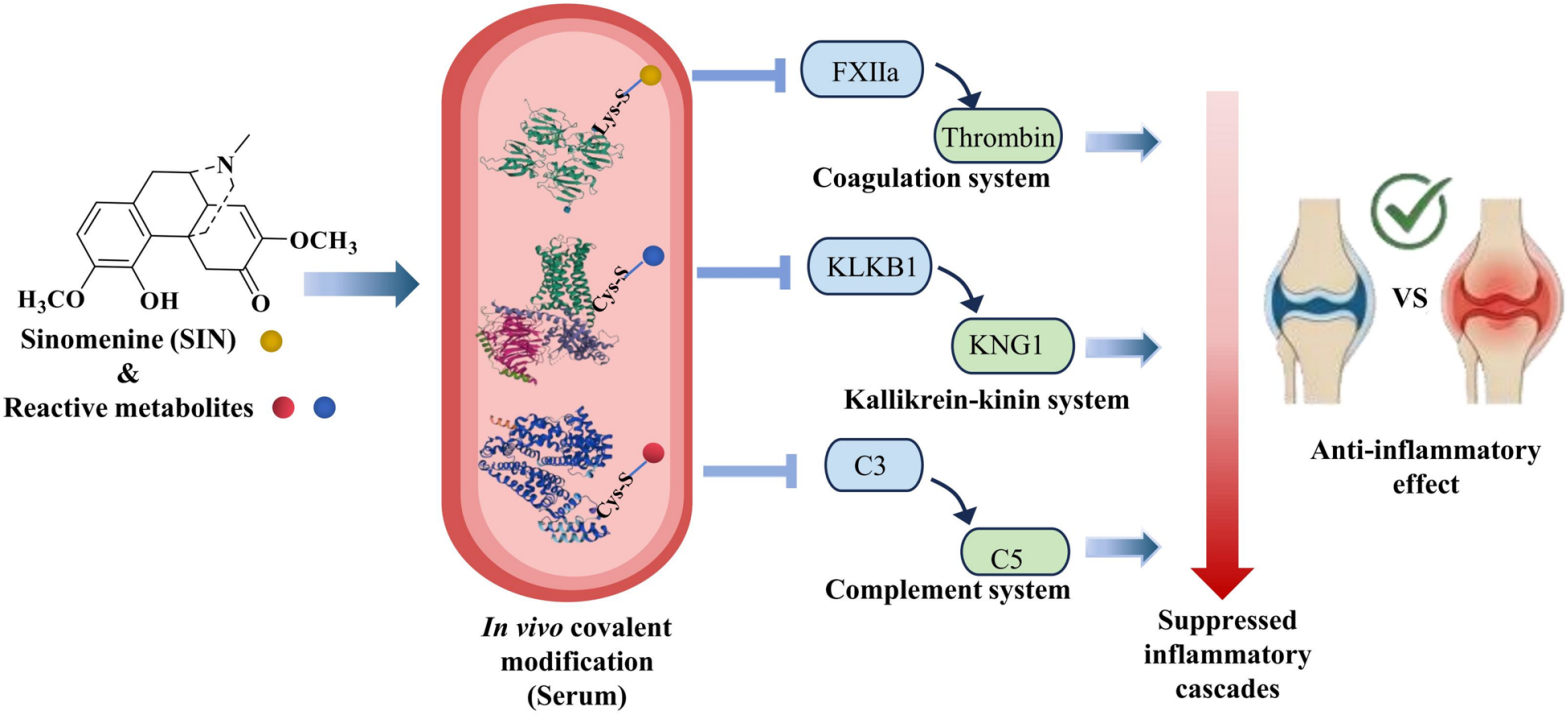

**Supplementary Information:**

The online version contains supplementary material available at 10.1186/s13020-026-01403-2.

## Introduction

Sinomenine (SIN) is a bioactive alkaloid isolated from *Sinomenium acutum* (Thunb.) Rehd. et Wils., a traditional Chinese medicinal plant that has been widely used in the treatment of inflammatory disorders, particularly rheumatoid arthritis (RA) [1]. In modern contemporary practice, SIN serves as the principal active ingredient of the approved Chinese patent medicine Zhengqing Fengtongning, demonstrating significant efficacy in alleviating joint inflammation and slowing disease progression in RA patients [2, 3]. Pharmacological studies have highlighted SIN’s anti-inflammatory and immunomodulatory capabilities, which include suppressing pro-inflammatory cytokines, inhibiting matrix metalloproteinase activity, and attenuating macrophage activation, thus helping to preserve joint structure and function [4–6].

Beyond its established role in RA, accumulating evidence suggests that SIN exhibits broader pharmacological activities across multiple disease contexts. Recent studies have reported its neuroprotective effects in models of diabetic cognitive dysfunction through modulation of oxidative stress-related signaling and the gut-brain axis [7]. In addition, SIN has shown antitumor potential in bladder cancer and glioma models by reversing multidrug resistance and promoting apoptosis [8, 9]. These diverse biological effects indicate that SIN acts through multiple molecular targets and signaling pathways, reflecting a complex and multifaceted mode of action. Although adverse reactions such as hypersensitivity have been reported in clinical settings [10–13], their relatively low incidence reinforces the overall therapeutic value of SIN, while emphasizing the need for a more nuanced understanding of its molecular mechanisms.

Covalent modification of proteins by drugs or their reactive metabolites (RMs) represents an important yet not fully understood aspect of pharmacological regulation. Such modifications may enhance therapeutic efficacy through sustained modulation of disease-related proteins, while also potentially contributing to adverse immune responses [14–16]. Notably, SIN contains an *α*,*β*-unsaturated ketone moiety, and its phase I metabolites, including oxygenated and demethylated forms, retain electrophilic properties capable of undergoing Michael addition reactions with nucleophilic amino acid residues such as cysteine and lysine [17, 18]. These chemical features raise the possibility that covalent protein modification may contribute to both the therapeutic actions and the complex biological behavior of SIN. However, the identities of the covalently modified protein targets and their functional relevance to inflammation-related pathways remain largely unexplored.

The systematic identification of drug-induced covalent protein modifications in biological systems presents substantial analytical challenges, primarily due to the low abundance and substoichiometric nature of these adducts. Traditional approaches, including nuclear magnetic resonance, radiolabeling, biotin-tagging strategies, and X-ray crystallography, have provided valuable insights but are often constrained by limited sensitivity, extensive sample requirements, or the need for prior knowledge of reactive sites [19–23]. In recent years, advances in mass spectrometry-based shotgun proteomics, together with improved peptide separation and data analysis strategies, have enabled more sensitive and unbiased detection of low-abundance protein modifications in complex biological matrices [24–26]. This aligns with the emerging “Chinmedomics” paradigm that integrates multi-omics technologies for comprehensive mechanism elucidation [27]. These developments provide new opportunities to characterize covalent drug-protein interactions in vivo.

**Fig. 1:**
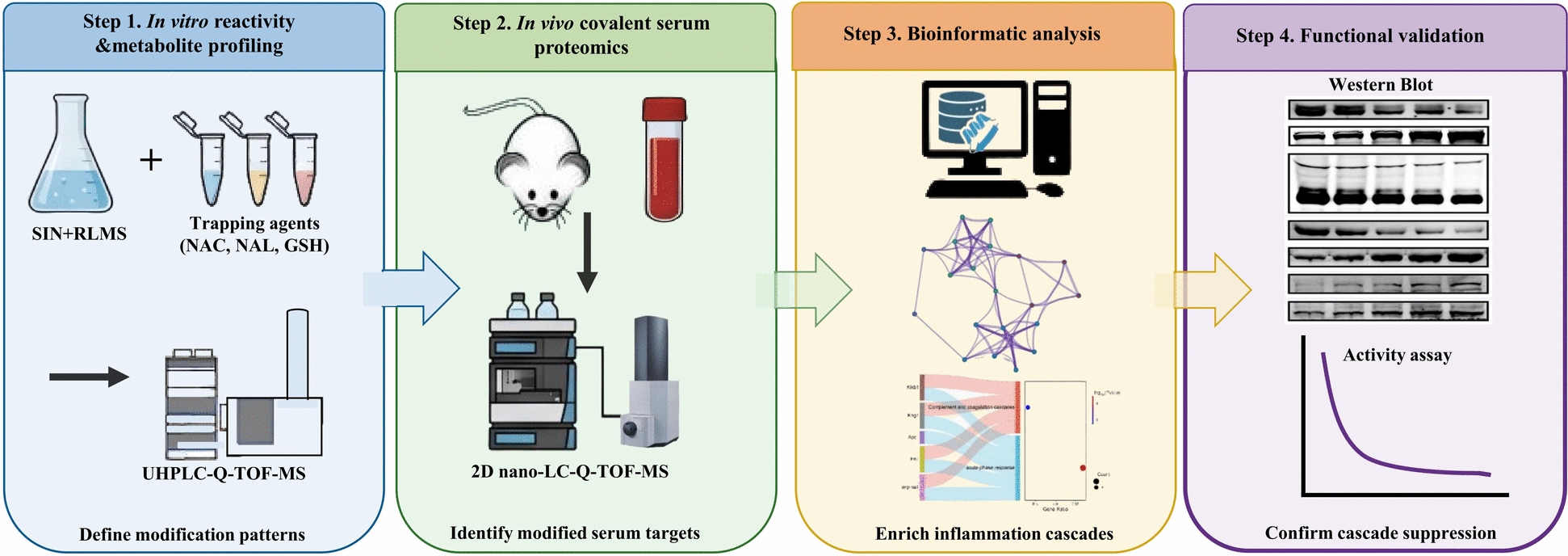
Schematic representation of the experimental workflow schematic. The study employed a four-stage approach: (1) In vitro reactivity profiling using rat liver microsomes (RLMs) and trapping agents (NAC, NAL, GSH) with UHPLC-Q-TOF-MS to define SIN’s modification patterns. (2) In vivo discovery using 2D nano-LC-Q-TOF-MS to identify covalently modified proteins in serum from SIN-treated rats. (3) Bioinformatic analysis to enrich inflammation-associated signaling cascades. (4) Functional validation using western blot and activity assays to confirm the suppression of key cascade components

In this study, we aim to unravel the underlying mechanisms of SIN’s roles in inflammation using a comprehensive proteomics-based approach coupled with liquid chromatography-mass spectrometry (LC-MS). Our strategy (Fig. 1) comprises several steps: initially, we utilize liver microsomes and model amino acids (AAs) with ultra-high-performance liquid chromatography coupled to quadrupole time-of-flight mass spectrometry (UHPLC-Q-TOF-MS) to elucidate potential modification patterns of SIN and its metabolites and identify potential modified amino acid residues. Subsequently, we employ two-dimensional nano-LC-Q-TOF-MS (2D nano LC-Q-TOF-MS) to isolate low-abundance modified peptides from rat serum, followed by database searching with Mascot incorporating the predicted modifications. Biological analyses of the identified SIN-modified proteins are then conducted using the STRING database and Metascape platform. Finally, western blot and activity assays are employed to validate SIN’s multi-target mode of action via covalent modification, elucidating its synergistic mechanisms in regulating inflammatory and related immune signaling pathways. This approach not only advances the understanding of SIN’s mechanism of action but also establishes a precedent for profiling covalent modifications induced by natural products, thus connecting traditional pharmacological knowledge with precision medicine approaches.

## Materials and methods

### Chemicals and reagents

Sinomenine (SIN) hydrochloride with a purity of over 98% was supplied by Tokyo chemical industry UK Ltd (TCI). NADPH generating solutions A and B and pooled rat liver microsome (RLM) were purchased from BD Gentest (San Jose, CA). Ammonium bicarbonate, *N*-acetyl lysine (NAL), *N*-acetyl cysteine (NAC), glutathione (GSH), and trifluoroacetic acid (TFA) were all obtained from Sigma-Aldrich (St. Louis, MO). Acetonitrile (ACN, HPLC grade) was provided by Anaqua Chemicals Supply Inc. Limited (Houston, TX). Dithiothreitol (DTT), iodoacetamide (IAA), and carbamide were purchased from GE Healthcare (Piscataway, NJ). Trypsin protease (MS grade) was obtained from Thermo Fisher Scientific (Rockford). The solid-phase extraction (SPE) C18 cartridge was from Waters Corporation (Milford, MA). The deionized water was prepared by a Millipore Milli-Q ultrapure water system (Millipore, Bedford, MA, USA). The ammonium persulfate (APS, BP179-100), tris base (BP152-5), Tween 20 (BP337-500) and glycine (BP381-5) were bought from Fisher Bioreagents (Fair Lawn, NJ, USA). The 30% acrylamide/bis solution (29:1, 1610156) and PVDF membranes (1620177) were obtained from Bio-Rad Laboratories (Hercules, CA, USA). The NuPAGE™ LDS sample buffer (4 × , NP0008) was purchased from Life Technologies (Carlsbad, CA, USA). The 2-mercaptoethanol (AC125472500) and *N*,*N*,*N*',*N*'-tetramethylethane-1,2-diamine (TEMED, AC433831000) was acquired from Acros Organics (Geel, Belgium). The sodium dodecyl sulfate (SDS, 18220) was purchased form Affymetrix (Cleveland, OH, USA). The factor XII (sc-59518) antibody was obtained from Santa Cruz Biotechnology (Santa Cruz, CA, USA). The plasma kallikrein 1B (KLKB1, ab1006), kininogen-1 (KNG1, ab175386), prothrombin (ab208590) antibodies were acquired from Abcam (Cambridge, MA, USA). The IRDye 800CW-conjugated goat anti-mouse IgG (926-32210) and goat anti-rabbit IgG (926-32211) secondary antibodies were purchased from LI-COR Biosciences (Lincoln, NE, USA). The complement component 3 (C3, AF6357) and complement component 5 (C5, AF6360) antibodies were obtained from Beyotime Biotechnology (Shanghai, China). The thrombin activity assay kit (AS-72129) was purchased from AnaSpec (Fremont, CA, USA).

### In vitro study with microsomes

#### SIN metabolism

The metabolic reaction was conducted by incubating SIN (0.55 mM) with RLM (1 mg/mL) in 100 mM phosphate buffer (pH 7.4) at 37 °C for 2 h, within a total system volume of 400 *µ*L containing NADPH-generating solution A (20 *µ*L) and solution B (4 *µ*L). SIN stock solution was prepared in water at a concentration of 44.32 mM for this purpose. At designated time points (0, 10, 20, 30, 60, 90, 120 and 240 min), 50 *μ*L were collected. Reaction termination was achieved by adding ice-cold ACN at a volume ratio of 5:1 (cold ACN to sample). The quenched samples were centrifuged at 15,000 g for 10 min. The clarified supernatants were then evaporated to dryness under nitrogen flow. The dry residues were dissolved in 50 *μ*L of a 50% methanol solution, centrifuged again, and the final solutions were analyzed by UHPLC-Q-TOF-MS analysis.

#### Co-incubation of SIN and AAs in RLMs

To assess the effects of amino acids on SIN metabolism, separate incubation systems were set up containing SIN (0.55 mM), RLM (1 mg/mL), and NADPH-generating solutions in 100 mM phosphate buffer (pH 7.4), with the addition of either NAL, NAC, or GSH (0.5 µM). All incubations were carried out at 37 ℃ for 1 h. For comparison, control groups were established by omitting either SIN or the amino acids from the complete reaction mixture. After incubation, the reaction was stopped with a fivefold volume of cold ACN. Subsequent sample processing involved centrifugation (15,000 g, 10 min), collection and nitrogen drying of the supernatant, and reconstitution of the residue in 50 *μ*L of 50% methanol. The prepared samples were centrifuged again prior to UHPLC-Q-TOF-MS analysis.

#### UHPLC-Q-TOF-MS/MS analysis

The separation and identification of SIN metabolites and SIN-AA adducts were achieved using UHPLC-Q-TOF-MS. The system consisted of an Agilent 1290 Infinity UHPLC unit interfaced with a 6550 Q-TOF-MS detector. The seperation was performed on an Agilent Eclipse XDB-C18 column (2.1 × 100 mm, 1.8 μm) housed in a thermostated compartment maintained at 40 ℃. The autosampler temperature was maintained at 4 ℃. A binary pump delivered the mobile phase at a flow rate of 0.3 mL/min with an injection volume of 1 *μ*L. Mobile phase A was water containing 0.1% formic acid, and mobile phase B was ACN with 0.1% formic acid. The gradient was as follows: 0–6 min, 5% B; 6–11 min, 5% → 15% B; 11–12 min, 15% → 95% B; 12–14 min, 95% B; 14–15 min, 5% B (equilibration: 3 min). MS detection was conducted in positive ion mode, scanning an *m/z* range 50–800. Key MS parameters included: dry gas flow, 15 L/min (250 ℃); sheath gas flow, 11 L/min (300 ℃); capillary voltage, 4000 V; nebulizer pressure, 25 psig; and nozzle voltage, 500 V.

### In vivo study

Male Sprague–Dawley (SD) rats weighing 200 ± 20 g were supplied by the Chinese University of Hong Kong. Upon arrival, the animals were acclimatized for seven days under standard laboratory conditions with a 12-h light/dark cycle, with free access to food and water. Prior to the experiment, the rats were subjected to a surgical procedure as previously described [28]. After a recovery period of at least 12 h, drug administration and blood collection were initiated. Blood sampling was conducted using an automated system, which included a robotic blood sampler (ABS2™, Instech, USA) and a freely moving rat containment device.

For the analysis of both drug-protein adducts and soluble metabolites, blood was collected from SD rats following SIN administration. Briefly, male rats (*n* = 3) were orally administered with SIN at 150 mg/kg (in 1 mL water), with a control group (*n* = 3) receiving water. Using an automated system, 300 *μ*L blood samples were collected at basedline (0 h) and 0.5, 1, 2 and 4 h post-dose. The samples underwent sequential centrifugation (3000 g and 14,000 g, 15 min each) to obtain clarified serum. Subsequently, 750 *µ*L ACN was added to the resulting supernatant to precipitate serum proteins. The protein precipitates were collected for targeted analysis of protein adducts using 2D nano LC-Q-TOF-MS Conversely, the corresponding supernatants were reserved for the profiling of SIN metabolites by UPLC-Q-TOF-MS.

#### Protein digestion and peptide purification

Protein digestion and subsequent peptide cleanup were performed as previously described [25]. Briefly, protein concentrations were normalized to 1 µg/*µ*L with Milli-Q water. Proteins were reduced with 200 mM DTT (1 h, 37 ℃), alkylated with 1 M IAA (1 h, 37 ℃ in the dark), and the reaction was quenched with an additional aliquot of DTT (1 h, 37 ℃). After dilution with 25 mM ammonium bicarbonate to reduce urea concentration below 1 M, tryptic digestion was carried out overnight (18 h, 37 ℃) at a 1:50 (w/w) enzyme-to-protein ratio. The resulting peptide digests were purified using 1 cc (50 mg) Sep-Pak cartridges (Waters). The cartridges were conditioned with 0.1% TFA in ACN and equilibrated with 0.1% TFA in water (6 × 1 mL each). Sample digests were loaded and washed with 0.1% TFA in water, and peptides were eluted with 200 *µ*L of 70:30 (v/v) ACN/water containing 0.1% TFA. The eluates were dried under nitrogen, reconstituted in 50 *μ*L 2:98 (v/v) ACN/water with 0.1% TFA, vortexed, centrifuged (13,000 g, 10 min), and stored at – 20 ℃ until analysis.

#### 2D nano LC-Q-TOF-MS/MS analysis

Peptide analysis was conducted using an integrated two-dimensional liquid chromatography system coupled to mass spectrometry. Briefly, 20 μg of SPE-purified peptides were injected into an UltiMate 3000 RSLC nano system (Thermo Scientific) interfaced with a Maxis Impact Q-TOF mass spectrometer (Bruker Corporation) equipped with a CaptiveSpray ion source. The first-dimensional separation utilized a strong cation exchange (SCX) column (PolySULFOETHYL A™, 100 mm × 0.3 mm, PolyLC Inc.). Peptides were fractionated by stepwise elution with increasing concentrations of ammonium acetate (5, 10, 25, 50, and 100 mM, pH 2.7) [29]. Each eluted fraction was automatically transferred and desalted on a peptide trap column (Acclaim PepMap, 2 cm × 75 μm, C18, Thermo Scientific) at 5 *μ*L/min using loading solvent (2% ACN, 0.1% formic acid). The second-dimensional separation was performed on a reversed-phase C18 analytical column (Acclaim PepMap RSLC, 15 cm × 75 μm, Thermo Scientific) at a flow rate of 300 nL/min with the following gradient: 5% B (0–30 min), 5–10% B (30–40 min), 10–35% B (40–130 min), 35–60% B (130–140 min), 60–80% B (140–145 min), and 80% B (145–150 min), where mobile phase A was 0.1% formic acid in water and B was 0.1% formic acid in ACN. The injection volume was 1 *μ*L. Mass spectrometry was operated in positive ion mode with the following parameters: dry gas temperature, 160 ℃; flow rate, 4.0 L/min; end plate offset, 500 V; and capillary voltage, 1400 V. Data-dependent acquisition was set to select the top 10 most intense precursors (*m/z* 300–1700, charge state >  + 1) for subsequent CID-MS/MS fragmentation.

#### Characterization of modified proteins

 Protein identification was conducted using Mascot against the Swiss-Prot 56.0 database. Variable modifications included the six potential SIN modification patterns (Table 2) and methionine oxidation, while cysteine carbamidomethyl (C) was selected as a fixed modification. Key search parameters included: MS/MS ion search (monoisotopic mass); mass tolerances of ± 30 ppm (precursor) and 0.2 Da (MS/MS); peptide charges (2 + , 3 + , and 4 +); and trypsin digestion (≤ 1 missed cleavage). Peptide-spectrum matches were validated by Percolator at a 1% false discovery rate (FDR), and protein identifications were accepted only when they met the Mascot significance threshold (*p* < 0.05). These criteria were applied to ensure the statistical confidence of peptide and protein identification.

### Biological function analysis of modified proteins

To elucidate the biological functions of the modified proteins, a systematic bioinformatics analysis was conducted. First, a protein–protein interaction (PPI) network was constructed using the STRING databas (https://string-db.org/) based on *Rattus norvegicus* protein identifiers. For network generation, only interactions with a combined confidence score of  ≥ 0.400 were retained, which integrates multiple lines of evidence including experimental data, curated databases, and text mining. Subsequently, the target protein list was submitted to the Metascape platform (https://metascape.org/) for functional enrichment analysis to identify significantly over-represented categories in Gene Ontology (GO) biological processes, cellular components, molecular functions, and KEGG pathways (significance threshold: *p* < 0.01 and enrichment factor > 1.5). Finally, to focus on specific biological themes, functional subnetworks were extracted from the global PPI network based on key GO terms derived from the enrichment analysis for visualization and in-depth interpretation.

### Determination of hematological effects by western blot assay

Three male SD rats were used in this experiment. Before treatment, 300 *μ*L of orbital blood samples were collected in anticoagulation tubes containing trisodium citrate. Subsequently, the rats were orally administered with 150 mg/kg SIN (dissolved in 1 mL water). At 0, 0.5, 1, 2 and 4 h post administration, 300 *μ*L of orbital blood samples were collected, respectively. The blood samples were centrifuged at 2500 g and 4 ℃ for 25 min to precipitate blood cells. The supernatant plasma was pipetted for western blot assay. Briefly, 10* μ*L of plasma was directly added with 90 *μ*L of sample loading buffer (NuPAGE™ LDS sample buffer, 1 × , containing 2% *β*-mecaptoethanol). After mixture and denaturation by boiling for 5 min, the plasma samples were subjected to gel separation, membrane transferring, immunoblotting with the primary and secondary antibodies and scanning by Odyssey infrared fluorescent scanner (LI-COR Biosciences). The pixel density of every band was quantified using ImageJ software (version 1.48v, NIH, Bethesda, MD, USA) for statistical analysis. The data were expressed as mean ± SD. The statistical analyses were used to compare the differences of protein levels at different time points.

### Determination of in vivo thrombin activity by fluorimetric analysis

The plasma from “Determination of Hematological Effects by Western Blot Assay” was also applied to evaluate the effect of SIN on in vivo thrombin activity. This experiment was conducted by fluorimetric analysis on the basis of the proteolytic cleavage of a synthetic substrate by thrombin and the subsequent release of a fluorophore, which could be measured by a fluorescence microplate reader. Briefly, 50 *μ*L of plasma from every sample was added to 96-well microplate (black, flat-bottom). Meanwhile, the equal volume of deionized water and gradient dilations of thrombin enzyme were used as negative and positive controls, separately. The microplate was pre-incubated at 37 ℃ for 10 min. Subsequently, 50 *μ*L of thrombin substrate solution was added into each well for another 30 min of incubation at 37 ℃. At last, after addition of 50 *μ*L of stop solution, the fluorescent intensity was measured by fluorescence microplate reader (SpectraMax iD5 Multi-Mode Microplate Reader, Molecular Devices, USA) with excitation and emission wavelengths of 490 nm and 520 nm, respectively. The relative thrombin activity of plasma was calculated on the basis of the linear regression equation from negative and positive controls. The data were expressed as mean ± SD. The statistical analyses were used to compare the differences of protein levels at different time points.

### Statistical analysis

Statistical analysis was performed using the software of GraphPad Prism 10.4.1 (GraphPad Software Inc., La Jolla, CA). Quantitative data were presented as the mean ± SD. Difference between two groups was analyzed using independent samples *t*-test. Statistical significance was defined as *p* < 0.05, with asterisks denoting significance levels: */#*p* < 0.05, **/##*p* < 0.01, ***/###*p* < 0.001.

## Results

### Chromatographic and MS/MS features of SIN

**Fig. 2:**
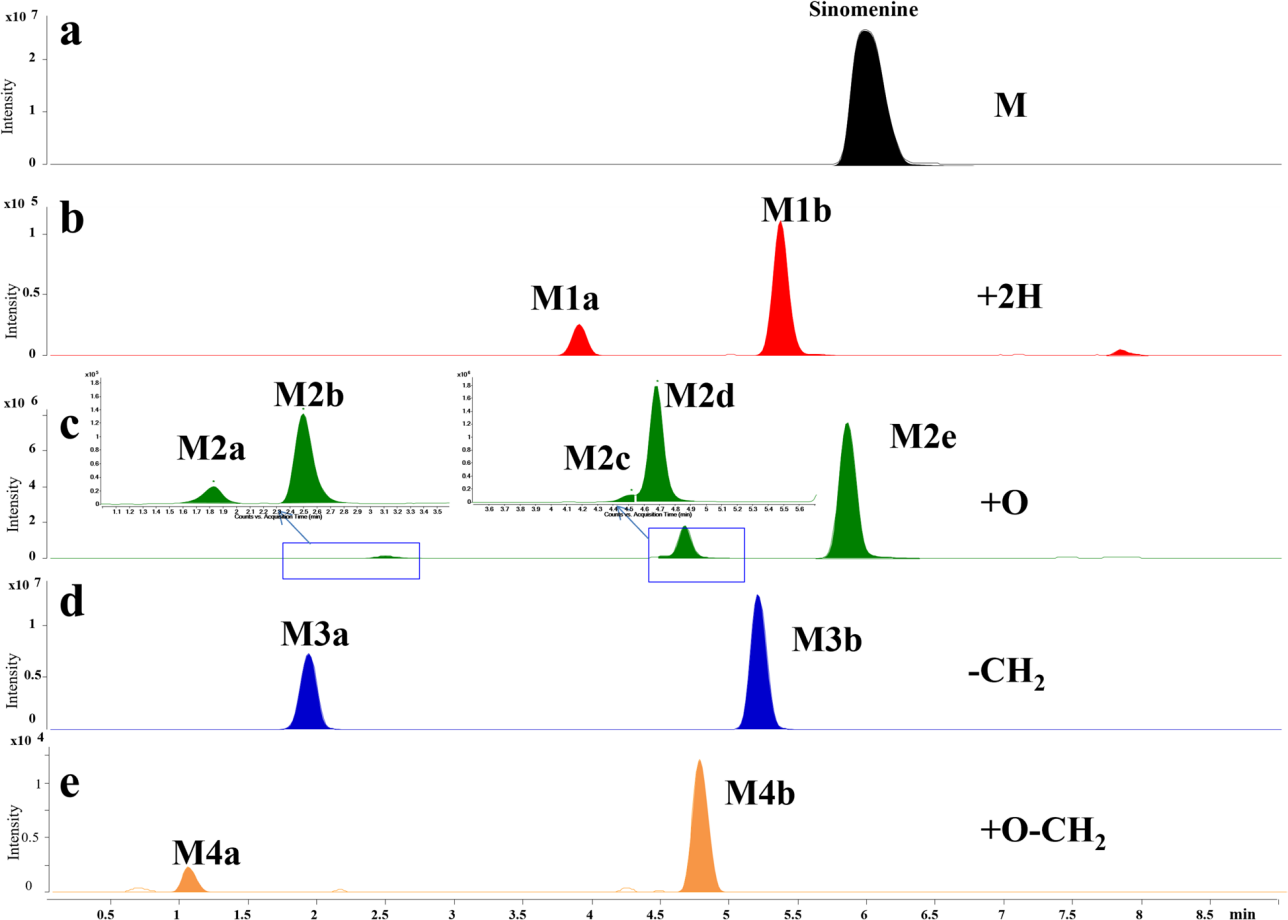
Metabolic profiles of SIN in rat serum. Extracted ion chromatogram (EIC) of SIN (**a**), SIN hydrogenated metabolites (**b**), oxygenated metabolites (**c**), demethylated metabolites (**d**) and oxygenated-demethylated metabolites (**e**)

To support confident assignment of SIN-derived metabolites and covalent conjugates, the chromatographic and fragmentation behaviors of SIN were first established. Under the current UHPLC conditions, SIN eluted as a single peak at 6.0 min (Fig. 2a). In positive mode, the MS/MS spectrum (Fig. S1) of SIN exhibited characteristic fragmentations involving loss of the piperidine ring [30]. Primary fragmentation pathways included neutral losses of methylamine (CH_3_NH_2_, Δm = 31) or amine moiety (CH_2_CHNHCH_3_, Δm = 57) from [M + H]^+^ ion [30, 31]. Additional fragment ions at *m/z* 267.1016, 239.1067 and 207.0804 were obtained by sequential losses of CH_3_OH, CO and 2CH_3_OH molecules from the [(M + H)-NH_2_CH_3_]^+^ ion separately. Ions at *m/z* 255.1016, 245.1172, 241.0859, 223.0754, 213.0910, 209.0597, 195.0804, 181.0648 and 153.0699 were generated by the continuous losses of H_2_O, CO or CH_3_OH from the [(M + H)-CH_2_CHNHCH_3_]^+^ ion as proposed in Fig. S1.

### Metabolite profiling of SIN in RLMs

To mimic in vivo metabolism, SIN was incubated with RLMs. After incubation with RLMs, 11 metabolites of SIN were determined using UHPLC-Q-TOF-MS (Table 1 and Fig. 2b–e). These metabolites could be grouped as hydrogenated metabolites (M1), oxygenated metabolites (M2), and demethylated metabolites (M3), and their structures were characterized by their chromatographic and MS/MS spectral behaviors in comparison with that of SIN (Fig. S2) as well as references [32, 33]. The detail information on the identification of these metabolites were put in Supporting information. Moreover, it was found that SIN metabolites peaked at 1 h after incubation at a concentration of 0.55 mM, therefore, this incubation time and concentration were selected in the following in vitro experiments.

**Table 1:** SIN metabolites detected in vitro

Compounds	Retention time (min)	Molecular formula	Calculated *m/z* [M+H]^+^	Measured *m/z* [M+H]^+^	Mass accuracy (ppm)	Increased mass (Da)
SIN	6.0	C_19_H_23_NO_4_	330.1700	330.1703	0.91	0
M1a	3.9	C_19_H_25_NO_4_	332.1856	332.1763	27.99	2.0157(+ 2H)
M1a	5.4	C_19_H_25_NO_4_	332.1856	332.1861	1.51	2.0157 (+ 2H)
M2a	2.1	C_18_H_23_NO_5_	346.1649	346.1617	9.24	15.9949 (+ O)
M2b	2.5	C_18_H_23_NO_5_	346.1649	346.1618	9.24	15.9949 (+ O)
M2c	4.5	C_18_H_23_NO_5_	346.1649	346.1614	10.11	15.9949 (+ O)
M2d	4.7	C_18_H_23_NO_5_	346.1649	346.1627	6.36	15.9949 (+ O)
M2e	4.9	C_18_H_23_NO_5_	346.1649	346.1617	9.24	15.9949 (+ O)
M3a	1.9	C_18_H_21_NO_4_	316.1543	316.1514	9.17	-14.0157 (-CH_2_)
M3b	5.3	C_18_H_21_NO_4_	316.1543	316.1521	6.96	-14.0157 (-CH_2_)
M4a	1.2	C_18_H_21_NO_5_	332.1492	332.1491	0.30	1.9792 (+ O-CH_2_)
M4b	4.9	C_18_H_21_NO_5_	332.1492	332.1490	0.60	1.9792 (+ O-CH_2_)

### Mapping covalent reactivity using amino-acid and peptide mimics in vitro

Two *N*-acetylated amino acids, *N*-acetylcysteine (NAC) and *N*-acetyllysine (NAL), were used as residue mimics to probe covalent reactivity in the presence of RLMs. In total, six NAC adducts and one NAL conjugate were detected (Table 2), supporting covalent reactivity toward both thiol- and amine-containing nucleophiles.

**Table 2:** SIN and its RMs conjugates formed with NAL, NAC, and GSH and the modification patterns

SIN adduct	Substrate	Retention time (min)	Molecular formula	Calculated *m/z* [M+H]^+^	Measured *m/z* [M+H]^+^	Mass accuracy (ppm)	Increased mass (Da)	Molecular composition of RM	Modification pattern
NAC1-1	NAC	1.3	C_24_H_30_N_2_O_8_S	507.1796	507.1803	1.38	343.1439	C_19_H_21_NO_5_	SIN-C1
NAC1-2	NAC	1.8	C_24_H_30_N_2_O_8_S	507.1796	507.1810	2.76	343.1439	C_19_H_21_NO_5_	SIN-C1
NAC1-3	NAC	3.2	C_24_H_30_N_2_O_8_S	507.1796	507.1809	2.56	343.1439	C_19_H_21_NO_5_	SIN-C1
NAC1-4	NAC	5.2	C_24_H_30_N_2_O_8_S	507.1796	507.1788	-1.58	343.1439	C_19_H_21_NO_5_	SIN-C1
NAC1-5	NAC	5.7	C_24_H_30_N_2_O_8_S	507.1796	507.1786	-1.97	343.1439	C_19_H_21_NO_5_	SIN-C1
NAC2	NAC	3.1	C_23_H_28_N_2_O_7_S	477.1687	477.1689	0.42	313.1314	C_18_H_19_NO_4_	SIN-C2
NAL	NAL	3.5	C_27_H_37_N_3_O_6_	500.2704	500.2760	11.19	311.1524	C_19_H_21_NO_3_	SIN-K1
GSH1	GSH	3.6	C_29_H_39_N_4_O_10_S	635.2381	635.2370	1.73	327.1471	C_19_H_21_NO_4_	SIN-C3
GSH2-1	GSH	1.3	C_29_H_38_N_4_O_11_S	651.2331	651.2331	0.00	343.1439	C_19_H_21_NO_5_	SIN-C1
GSH2-2	GSH	3.6	C_29_H_38_N_4_O_11_S	651.2331	651.2328	0.46	343.1439	C_19_H_21_NO_5_	SIN-C1
GSH3-1	GSH	1.6	C_28_H_36_N_4_O_10_S	621.2225	621.2200	4.02	313.1314	C_18_H_19_NO_4_	SIN-C2
GSH2-2	GSH	4.8	C_28_H_36_N_4_O_10_S	621.2225	621.2218	1.13	313.1314	C_18_H_19_NO_4_	SIN-C2

#### NAC conjugates

**Fig. 3:**
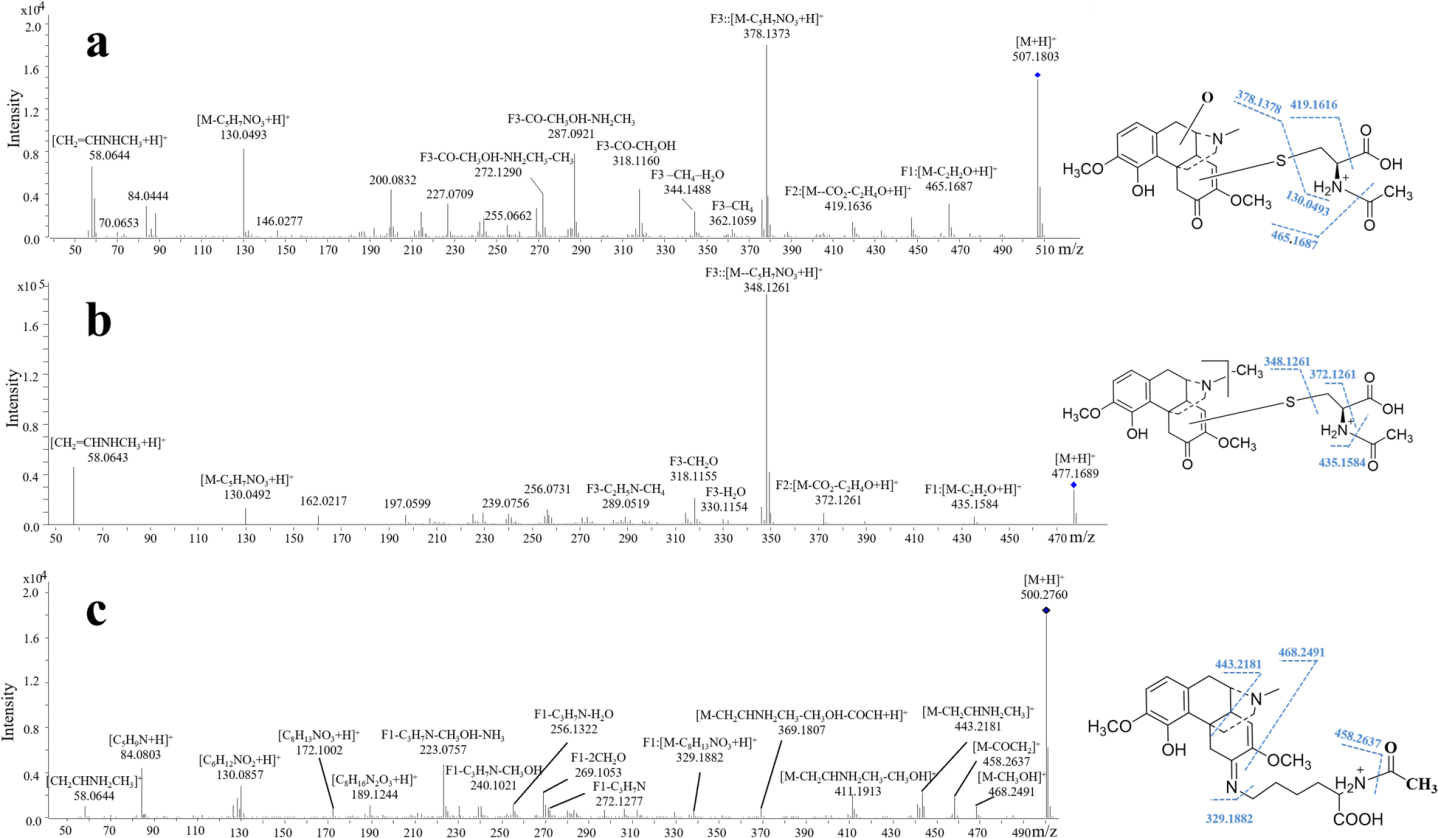
MS/MS spectra and possible structures of SIN and its RMs conjugates with NAC (**a** NAC1; **b** NAC2) and NAL (**c**)

The adducts NAC1-1 (Rt = 1.3 min), NAC1-2 (Rt = 1.8 min), NAC1-3 (Rt = 3.2 min), NAC1-4 (Rt = 5.2 min) and NAC1-5 (Rt = 5.7 min) generated a protonated molecule at *m/z* 507.1796 (Fig. S3a). Accurate mass measurement given the chemical formula of C_24_H_30_N_2_O_8_S, suggesting that NAC was added to oxygenated SIN (M2). Their same fragment ions of *m/z* 465.1687 (-C_2_H_2_O), *m/z* 419.1636 (-CO_2_-C_2_H_4_O), *m/z* 378.1373 (-C_5_H_7_NO_3_) and *m/z* 130.0493 (C_5_H_7_NO_3_) were also proved the addition of NAC. Ions of *m/z* 362.1059, 344.1488, 318.1160, 287.0921, 272.1290, 255.0662, 227.0709 and 58.0644, which were generated from NAC1 due to a series loss of CH_4_, H_2_O, CO, CH_3_OH, NH_2_CH_3_ and CH_2_=CHNHCH_3_, were similar to the fragmentation behavior of oxygenated SIN (M2) (Fig. 3a). NAC2 (Rt = 3.7 min) given a [M + H]^+^ at *m/z* 477.1690 with a chemical formula of C_23_H_28_N_2_O_7_S (Fig. S3b). The ions of *m/z* 435.1584 (-C_2_H_2_O), *m/z* 372.1261 (-CO_2_-C_2_H_4_O), *m/z* 348.1261 (-C_5_H_7_NO_3_) and *m/z* 130.0492 (C_5_H_7_NO_3_) indicating that NAC was added to demethylated SIN (M3) (Fig. 3b).

#### NAL conjugate

A single NAL conjugate (Rt = 3.5 min) was observed with [M + H]^+^ at *m/z* 500.2704 with a chemical formula of C_27_H_37_N_3_O_6_ (Fig. S3c). The fragment ions of *m/z* 468.2491 (-CH_3_OH), 458.2637 (-COCH_2_), 443.2181 (CH_2_CHNH_2_CH_3_), 411.1913 (CH_2_CHNH_2_CH_3_-CH_3_OH), 369.1807 (-CH_2_CHNH_2_CH_3_-CH_3_OH-COCH) and 329.1882 (-C_8_H_13_NO_3_) suggested that the NAL was added to SIN and the carbanyl group in C6 of SIN was substituted by the terminal -NH_2_ group in NAL. This was further conformed by the ions of *m/z* 272.1277, 256.1322, 240.1021, 223.0575, 172.1002 and 130.0857, as displayed in Fig. 3c.

#### GSH conjugates

GSH was used as a peptide-level nucleophile, and five GSH conjugates were detected. GSH1 (Rt = 3.6 min, Fig. S3d) generated a protonated molecule at *m/z* 635.2381 and accurate mass measurement given the chemical formula of C_29_H_39_N_4_O_10_S. As shown in Fig. S4a, fragment ions by the loss of C_5_H_7_NO_3_ (*m/z* 506.1923) and C_10_H_15_N_3_O_6_ (*m/z* 362.1429) were observed in MS/MS spectra. The loss ions contained an amino group provided the evidence that GSH bound to SIN through the sulfhydryl group. Therefore, GSH1 was tentatively identified as GSH-SIN conjugate via Michael Addition. In the same way, GSH2 adducts (*m/z* 651.2331, C_29_H_38_N_4_O_11_S) were supposed to be GSH-oxygenated SIN conjugates (Fig. S3e, Fig. S4b), and GSH3 adducts (*m/z* 621.2225, C_28_H_36_N_4_O_10_S) were reasonably identified as GSH-demethylated SIN conjugates **(**Fig. S3f, Fig. S4c) based on their MS behaviors. In addition, conjugates GSH2 and GSH3 adducts all generated an ion at *m/z* 184.0740 (-C_9_H_13_NOS) in their MS/MS spectra, which further confirmed that GSH was added to SIN and its RMs via the sulfhydryl group in GSH.

Collectively, these trapping experiments shown that SIN and its metabolites had the potential to modify AA residues in proteins via cysteine or lysine groups with three different forms (Table 2). Considering the AA residues of cellular proteins might be modified by SIN and its metabolites in the same way. Then, in vivo experiments were conducted, and the molecular formulas of SIN and its metabolites were added into Mascot as a variable modification in advance.

### Identification of SIN and its metabolite-modified proteins in vivo

Serum was collected after SIN administration, and serum proteins from a selected post-dose time point were analyzed by 2 D nano-LC-Q-TOF-MS to identify covalently modified peptides.

The assignment of SIN-modified peptides was based on the integrated evaluation of multiple established proteomic criteria, including accurate precursor mass shift, informative backbone b/y ions, residue-level site-localizing fragment ions, and high-confidence peptide-spectrum matches under stringent FDR control. To further improve confidence and transparency, the low-*m/z* regions of all corresponding MS/MS spectra were manually re-examined. In addition, relative extracted ion chromatogram comparisons between SIN-treated and control samples showed that the identified SIN-modified peptides were preferentially detected in SIN-exposed animals, whereas little to no corresponding signal was observed in controls, further supporting their treatment-associated occurrence (Fig. S7).

#### Rat serum albumin adducts

**Fig. 4:**
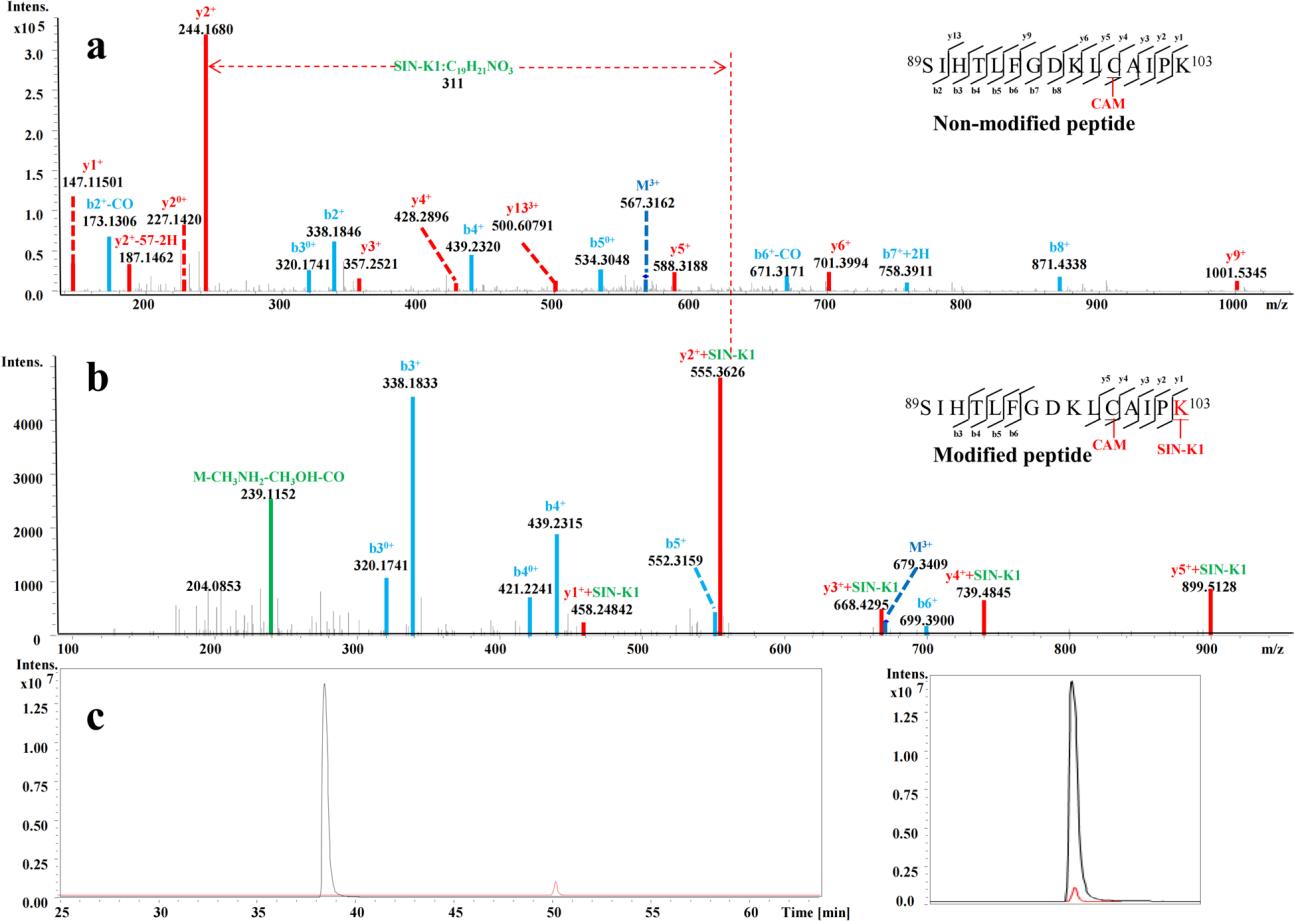
MS/MS spectra for SIN RM-modified peptide (**b**) and non-modified peptide (**a**) of SIHTLFGDKLCAIPK in RSA and their extraction ion chromatograms (EIC), Black trace: extracted ion chromatogram for the unmodified peptide, Red trace: extracted ion chromatogram for the SIN-modified peptide (**c**). SIN-related fragment ions are highlighted in green. CAM represents as modified by carbamidomethylating

Four albumin-derived peptides were identified as modified at lysine or cysteine residues by SIN or its metabolites (Table 3). For example, peptide ^89^SIHTLFGDKLCAIPK^103^ exhibited a quasi-molecular ion [M + 2H]^2+^ at *m/z* 679.3409 (Fig. 4b), an addition of 311 Da compared with the corresponding non-modified peptide (Fig. 4a). The fragmentation ions of y1, y2, y3, y4 and y5 were also found to increase 311 Da than that of the non-modified peptide; therefore, the lysine should be modified by SIN, with the carbonyl group in C6 of SIN substituted by the terminal -NH_2_ group in lysine. The fragmentation ion *m/z* 239 from SIN was further confirmed the modification pattern, while other fragmentation ions, b3-b6, indicated that the peptide belonged to ^89^SIHTLFGDKLCAIPK^103^. EIC indicated that the MS intensity of the modified peptide was evidently lower than that of the nonmodified peptide (Fig. 4c), consistent with the substoichiometric nature typical of xenobiotic-induced covalent adducts. This low abundance also underscores the importance of effective chromatographic separation for reliable MS/MS acquisition. Nevertheless, even low-level modification of key functional proteins may yield measurable biological consequences if the affected proteins occupy critical regulatory positions within protease or inflammatory cascades [34]. The modified peptide of ^528^AETFTFHSDICTLPDK^543^ exhibited a quasi-molecular ion [M + 2H]^3+^ at *m/z* 713.3349, which was increased 313 Da than that of the corresponding non-modified peptide (Fig. S5a). The fragmentation ions of y6 and y8-y14 were also found to increase 313 Da than that of the non-modified peptide, while other fragmentation ions, b2-b4, b6, b9 and b10 indicated that the peptide belonged to ^528^AETFTFHSDICTLPDK^543^. Therefore, the cystine should be modified by demethylated SIN (SIN-C2). In the same way, the cysteine in peptide ^525^EFKAETFTFHSDICTLPDK^543^ (Fig. S5b) and ^282^AELAKYMCENQATISSK^298^ (Fig. S5c) were found to be adducted by modification pattern SIN-C2 and SIN-C3 separately (Table 3). Control samples processed in parallel without SIN yielded no corresponding adducted peptides, supporting SIN-dependence of these observations.

**Table 3:** Identified SIN RM-Protein Adducts in rat serum

Proteins	Entry	Modified peptide	Sequence positions	Modified site	*m/z* (charge) observed	Mass accuracy (ppm)	Adducts	RT (min)
Rat serum albumin (RSA)	P02770	SIHTLFGDKLCAIPK	89–103	–	567.3162(3 +)	21	None modification	40.1
		SIHTLFGDKLCAIPK	89–103	K103	679.3409(2 +)	22	SIN-K1	50.2
		EFKAETFTFHSDICTLPDK	525–543	C538	636.5578 (4 +)	14	SIN-C2	40.5
		AETFTFHSDICTLPDK	528–543	C538	713.3349(3 +)	5	SIN-C2	35.5
		AELAKYMCENQATISSK	282–298	C289	744.0105(3 +)	11	SIN-C1	21.5
Serum amyloid P component (SAP)	P23680	DKVGQYSLYIGNSK	86–99	K99	941.9732 (2 +)	10	SIN-K1	41.5
Alpha-1-inhibitor III (A1i3)	P14046	ISLCHGNPTFSSETKSGCK	266–284	C269	799.3771 (3 +)	11	SIN-C2	49.4
Alpha-1-antiproteinase (A1AT)	P17475	MQHLEQTLTK	278–287	K287	513.9471 (3 +)	20	SIN-K1	20.3
Kininogen-1 (KNG1)	P08934	NNKFSIATQICNITPGK	116–132	C126	726.0487 (3 +)	10	SIN-C3	114.6
Fibronectin (FINC)	P04937	TFYQIGDSWEK	568–578	K578	562.2525 (3 +)	-28	SIN-K1	33.8
Plasma kallikrein (KLKB1)	P14272	CLLFSFLAVSPTK	57–69	C57	884.9390 (2 +)	-30	SIN-C1	43.9

#### Adducts on functional serum proteins

**Fig. 5:**
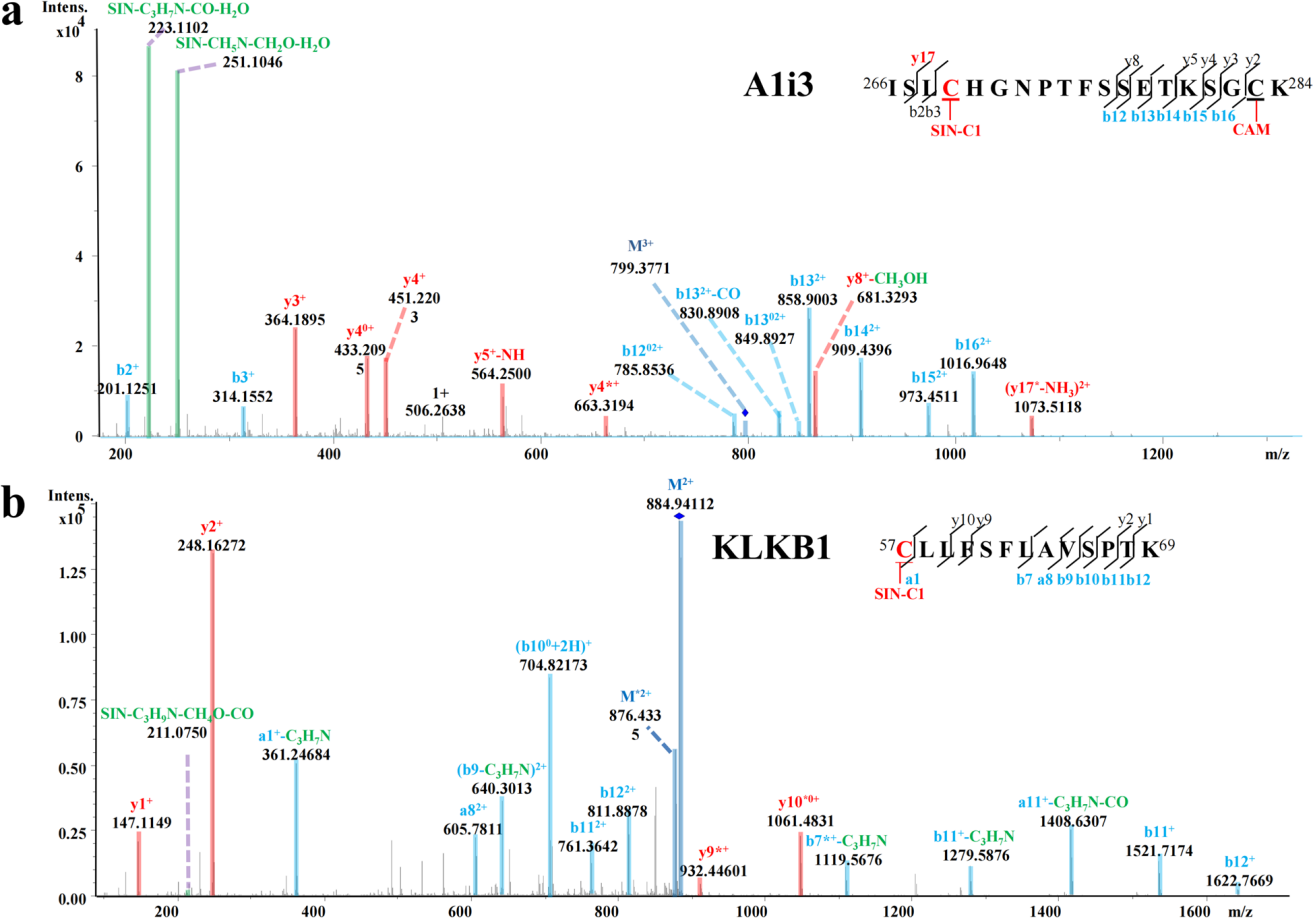
MS/MS spectra of SIN RM-adducted peptides for protein A1i3 (**a**) and KLKB1 (**b**). SIN-related fragment ions are highlighted in green. CAM represents as modified by carbamidomethylating

In addition to albumin, six additional serum proteins were identified as covalently modified (Table 3), yielding a total of seven SIN-modified serum proteins. These included alpha-1-inhibitor III (A1i3), plasma kallikrein (KLKB1), kininogen-1 (KNG1), alpha-1-antitrypsin (A1AT), fibronectin (FINC), and serum amyloid P component (SAP). Both A1i3 and KLKB1 were modified by the metabolite SIN-C1, while KNG1 was modified by SIN-C3, all at specific cysteine residues. The quasi-molecular ion [M + 3H]^2+^ at *m/z* 799.3771 matched the adducted peptide of ^266^ISLCHGNPTFSSETKSGCK^284^ for A1i3 and the mass increase of 343 Da compared to the non-adducted peptide suggested the modification of SIN-C1. The fragmentation ions of y17 and b12-b16 were also found to increase by 343 Da compared to the un-aged peptide. Therefore, the binding site should be C269 (Fig. 5a), while other fragmentation ions, b1, b3, y2-y5 and y8, observed no significant difference. This kind of modification pattern was also observed in the KLKB1 (Fig. 5b). Although the direct SIN-derived ion in the KLKB1 spectrum was of low intensity and therefore less visually prominent, additional SIN-related characteristic fragment ions, including ions consistent with neutral loss or partial loss of SIN-associated moieties such as C_3_H_7_N, were still detectable, providing further support for the assignment of SIN-C1 modification. In contrast, a distinct SIN-C3 modification was detected in KNG1. The quasi-molecular ion [M + 3H]^3+^ at *m/z* 726.0487 matched the aged peptide of ^116^NNKFSIATQICNITPGK^132^ for KNG1, and the addition of 327 Da to the non-adducted peptide indicted the modification of SIN. The fragmentation ion of b11 was also observed to increase 327 Da compared with that of the unaged peptide. Hence, the binding site was supposed to be C126 (Fig. S6c). Although no clear SIN-related low-*m/z* ions were identified for this peptide, the assignment was supported by accurate precursor mass shift, informative backbone b/y ions, residue-level site-localizing fragment ions, and high-confidence peptide-spectrum matching under stringent FDR control.

Lysine-centered modification was also observed. A1AT peptide ^278^MQHLEQTLTK^287^ showed a molecular ion [M + 3H]^3+^ at *m/z* 513.9471, which was increased 311 Da than that of the corresponding unmodified peptide; thus, it should be modified by SIN-K1. The modification site was thought to be K287 from the same mass addition of 311 Da to the fragment ions of y2-y9 of the aged peptide compared with the unaged peptide (Fig. S6a). Both the direct SIN-derived ion and SIN-related characteristic fragments were detected in A1AT. Similar phenomenon were observed for Fibronectin (FINC) (Fig. S6b) and serum amyloid P component (SAP) (Fig. S6c), in which direct SIN-derived ions were not confidently detected, whereas SIN-related loss ions remained observable and supported the proposed modifications.

### Pathway association and functional readouts of coagulation, kallikrein-kinin and complement regulation

**Fig. 6:**
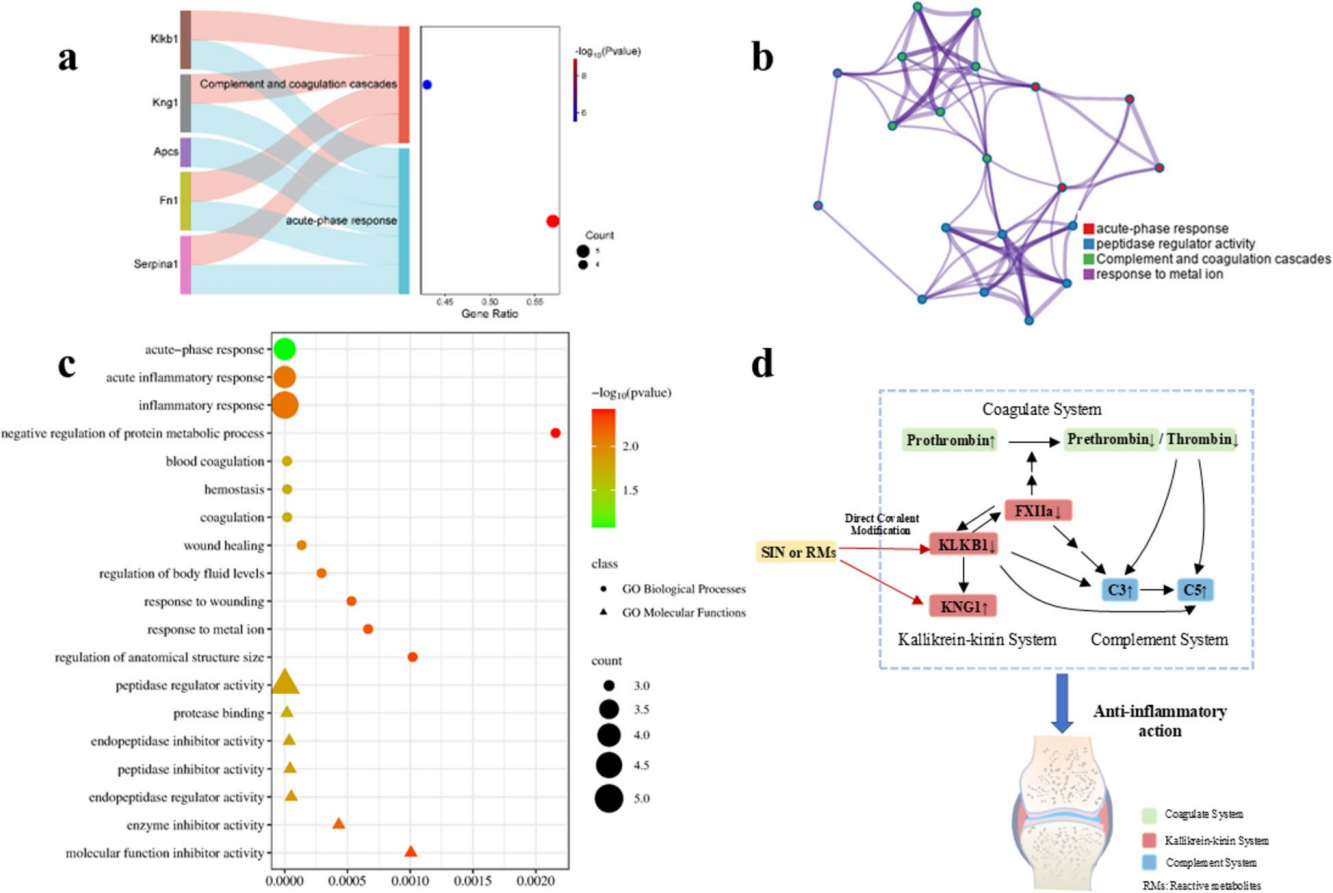
Biological analysis of SIN target proteins in rat serum using STRING and Metascape database platform. **a** KEGG pathway enrichment results, *p* < 0.001. **b** Network of enriched terms: colored by cluster ID, where nodes that share the same cluster ID are typically close to each other. **c** GO enrichment analysis of the biological process and molecular functions, *p* < 0.001. **d** The systems regulated by SIN and the relationship with anti-inflammation effects

Proteomic analysis identified several proteins covalently modified by SIN and its RMs. To bridge the identification of these modified proteins and their biological function, we performed a bioinformatic enrichment analysis. This analysis revealed that the modified proteins are predominantly involved in complement and coagulation cascades and acute-phase response (Fig. 6a–c). These pathways collectively form a triad of inflammation-amplifying circuits within the circulatory system. Therefore, the effects of SIN on the expression of these proteins are worthy of further study.

**Fig. 7:**
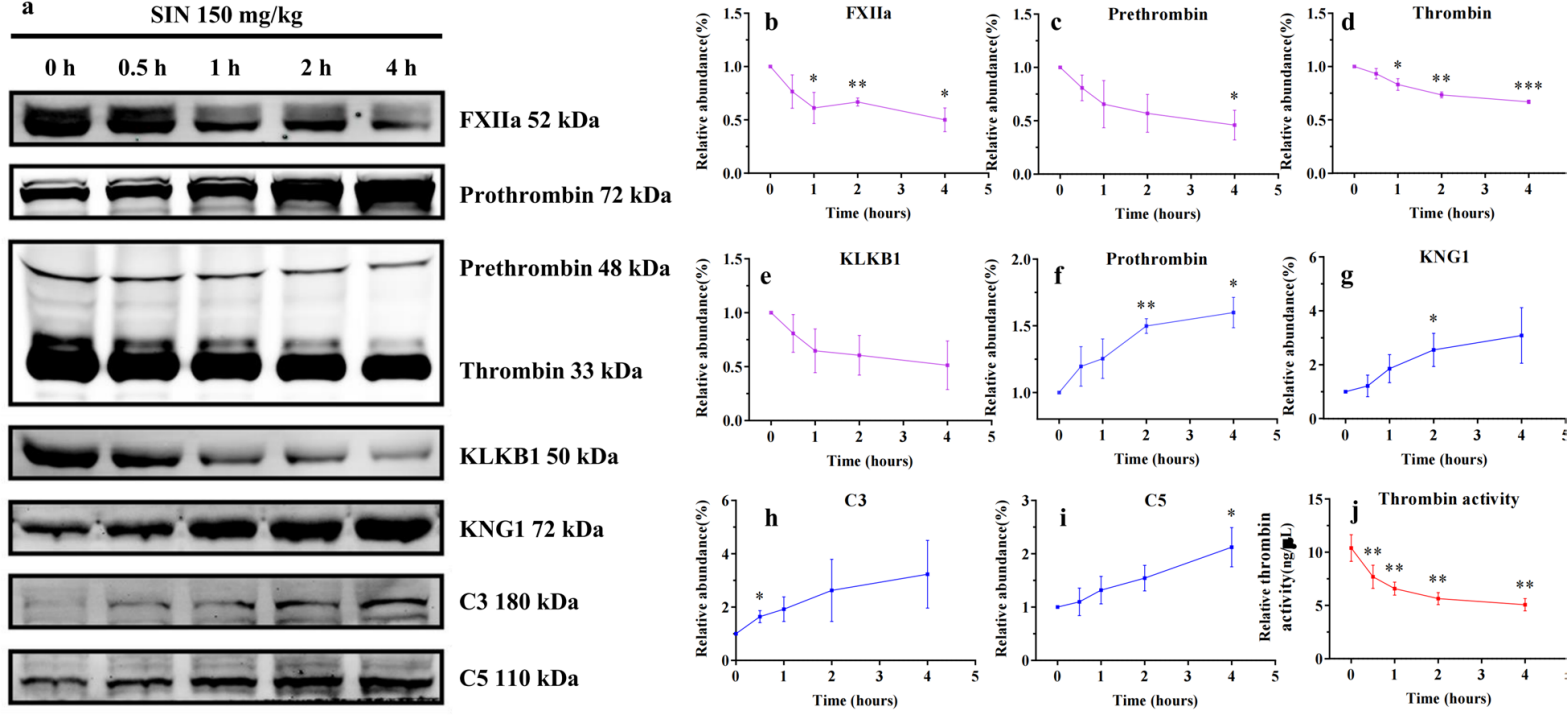
SIN exerts in vivo anti-inflammation effects by inhibiting the crosstalk of the kallikrein-kinin, complement, and coagulation systems. **a** SIN suppresses the key proteins of kallikrein-kinin, complement, and coagulation systems in a time-dependent manner. SIN-treated rat plasma was subjected to western blot assay. Representatives immunoblot figures are shown. **b**–**i** The relative abundance of these key proteins was quantified by ImageJ. **j** SIN inhibits the activity of thrombin in a time-dependent manner. All data were from three rats. Statistical analyses were conducted by GraphPad Prism. *p < 0.05, **p < 0.01 and ***p < 0.001, compared with vehicle control (0 h)

To assess whether these target associations were accompanied by pathway-level changes, time-dependent western blotting of rat plasma post-SIN administration quantified key effectors: activated FXII (FXIIa 52 kDa), prothrombin (72 kDa), prethrombin (48 kDa), thrombin (33 kDa), KLKB1 (50 kDa), KNG1 (72 kDa), C3 (180 kDa) and C5 (110 kDa). As results, SIN strongly downregulated the abundances of FXIIa, prethrombin, thrombin and KLKB1 (Fig. 7a–e), and increased the content of prothrombin, KNG1, C3 and C5 (Fig. 7f–i) in a time-dependent manner. A fluorometric assay confirmed a time-dependent reduction of thrombin activity in plasma following SIN treatment (Fig. 7j). Based on the above results, SIN could triger the cascade inhibition of coagulation, kallikrein-kinin and complement systems, so as to decrease the inflammatory response (Fig. 6d).

## Discussion

This study explores the potential role of covalent protein modification in the anti-inflammatory activity of SIN. By integrating metabolite profiling, in vitro nucleophile trapping, in vivo serum proteomics and functional validation, we showed that SIN and its metabolites are capable of covalently modifying serum proteins and that the identified targets are closely associated with inflammation-related proteolytic cascades.

The in vitro trapping experiments using NAC, NAL and GSH indicated that SIN-derived electrophilic species could react with nucleophilic residues characteristic of cysteine and lysine. These findings were consistent with the chemical features of SIN and its phase I metabolites, which retain an *α*,*β*-unsaturated carbonyl moiety susceptible to Michael-type addition reactions [17, 18]. Importantly, the identification of multiple conjugation patterns suggests that covalent interactions are not restricted to a single reaction mode, supporting the feasibility of diverse protein adduct formation under metabolically competent conditions.

The in vivo serum proteomics analysis indicated that SIN or its metabolites modified proteins were not randomly distributed but instead converged on circulating pathways that play central roles in inflammatory amplification. In addition to serum albumin, which likely reflects systemic exposure and the feasibility of covalent binding in plasma, several biologically relevant targets were identified, including KLKB1, KNG1, A1i3, A1AT, FINC, and SAP. These proteins are functionally linked to the interrelated kallikrein-kinin, coagulation, and complement systems, consistent with the pathway enrichment analysis. Among them, KLKB1and KNG1 are core components of the kallikrein-kinin system and are directly involved in bradykinin generation, a critical process in inflammation, pain, and vascular permeability [35–37]. A1i3 and A1AT, as regulators of serine protease activity, suggest that SIN-associated covalent modification may also influence protease-antiprotease balance, which is closely linked to inflammatory tissue injury and coagulation-complement crosstalk [38–40]. In addition, FINC and SAP further support the inflammatory relevance of the covalent target landscape, given their established roles in extracellular matrix-associated inflammation, innate immune regulation, and complement-related acute-phase responses [39, 41, 42]. Collectively, these findings suggest that SIN targets a highly interconnected inflammatory network rather than isolated proteins.

To define the functional consequences of SIN-induced covalent modification, we analyzed key nodes within the kallikrein-kinin, coagulation, and complement pathways. Within the kallikrein-kinin system, KLKB1 and KNG1 form a canonical enzyme-substrate pair, in which KLKB1 cleaves KNG1 to liberate a biologically active peptide, bradykinin [43]. Thus, covalent modification of KLKB1 and KNG1 by SIN would be expected to impair the catalytic process so as to reduce KNG1 cleavage, which consequently brought about the marked accumulation of KNG1 observed in the western blot assay. FXIIa can catalyze the conversion of plasma prekallikrein (PPK) to KLKB1, and KLKB1 could in turn amplifies FXII activation via a positive feedback loop [44]. Accordingly, the inhibitory effect of SIN on KLKB1 by covalent modification reduced FXIIa levels as well as the ensuing KLKB1 contents shown in western blot assay. Decreased FXIIa also repressed the activation of coagulation system, as reflected by increased prothrombin, decreased prethrombin and thrombin, and reduced thrombin activity. This inhibitory effects propagated downstream to complement system by reduced FXIIa and thrombin, both of which inhibited complement activation as indicated by increased C3 and C5 levels. Collectively, these findings reveal a previously underappreciated mechanism in which SIN may modulate inflammation-associated proteolytic networks through covalent modification. While indirect regulatory mechanisms cannot be entirely excluded, the consistency of these pathway-level changes with established biochemical principles supports covalent modification as a central driver of the observed effects. Nevertheless, further investigations, such as site-directed mutagenesis, biochemical assays with purified target proteins, and time-resolved analyses, are warranted to directly establish the functional contribution of specific modification events.

From a methodological perspective, the identification of low-abundance covalently modified peptides in serum highlights the utility of combining two-dimensional nano-LC separation with high-resolution mass spectrometry for in vivo covalent proteomics. Given the sub-stoichiometric nature of covalent adducts in complex biological matrices, further methodological advances, such as targeted enrichment or complementary fragmentation strategies, may expand coverage of covalent targets. Nevertheless, the present workflow established a feasible strategy for profiling covalent protein modification induced by electrophilic natural products.

In summary, this study supports a model in which SIN and its metabolites can covalently modify serum proteins at cysteine or lysine residues through multiple reaction modes. The identified in vivo targets were enriched in coagulation, kallikrein-kinin and complement-related pathways, and functional assays were consistent with coordinated suppression of these interconnected inflammatory cascades. These findings provide evidence of a potential mechanistic link between SIN-induced covalent protein modification and its anti-inflammatory activity. Importantly, this study highlights covalent proteomics as a valuable approach for elucidating the molecular basis of bioactive compounds derived from traditional Chinese medicine.

## Supplementary Information

Supplementary Material 1.

## Data Availability

All data generated or analyzed during this study are included in this published article (and its supplementary information files).

## Abbreviations

SIN: Sinomenine
RA: Rheumatoid arthritis
LC-MS: Liquid chromatography-mass spectrometry
UHPLC-Q-TOF-MS: Ultra-high-performance liquid chromatography coupled to quadrupole time-of-flight mass spectrometry
2D nano LC-Q-TOF-MS: Two-dimensional nano-LC-Q-TOF-MS
RLM: Rat liver microsome
ACN: Acetonitrile
NAC: N-acetyl cysteine
NAL: N-acetyl lysine
GSH: Glutathione
TFA: Trifluoroacetic acid
DTT: Dithiothreitol
IAA: Iodoacetamide
SPE: Solid-phase extraction
TEMED: *N*,*N*,*N*',*N*'-Tetramethylethane-1,2-diamine
SDS: Sodium dodecyl sulfate
FDR: False discovery rate
PPI: Protein-protein interaction
GO: Gene Ontology
EIC: Extracted ion chromatogram
RSA: Rat serum albumin
KLKB1: Plasma kallikrein
KNG1: Kininogen-1
A1i3: Alpha-1-inhibitor III
A1AT: Alpha-1-antitrypsin
FINC: Fibronectin
SAP: Serum amyloid P component
FXIIa: Activated FXII
C3: Complement component 3
C5: Complement component 5
WB: Western blot
RMs: Reactive metabolites
AAs: Amino acids
SCX: Strong cation exchange
CAM: Carbamidomethylating
KKS: Kallikrein-kinin system
PPK: Plasma prekallikrein
