# Supplementary material for: Chemical proteomics reveals sinomenine’s anti-inflammatory mechanism through serum protein covalent modification
Source: Chin Med. 2026 Apr 30;21:124. doi: 10.1186/s13020-026-01403-2 (PMC13130404; doi:10.1186/s13020-026-01403-2)
Supplement: Supplementary file 1 — Supplementary Material 1. [file 13020_2026_1403_MOESM1_ESM.doc]

Supporting Information

Tittle

**Chemical Proteomics Reveals Sinomenine’s Anti-inflammatory Mechanism through Serum Protein Covalent Modification**

**Author(s), and Corresponding Author(s)***

Meixian Liu^1,2,＃^, Zhiyuan Zheng^1,3,＃^, Yida Zhang^1,4^, Xiqing Bian^5^, Yue Zhuo^1,6^, Hai-Ying Wu^7^, Jian-Lin Wu^1^,*, Na Li^1^,*

**Affiliations**

^1^State Key Laboratory of Mechanism and Quality of Chinese Medicine, Macau University of Science and Technology, Avenida Wai Long, Taipa, Macau 999078, China

^2^ranslational Innovation Center, Shenzhen Bay Laboratory, Shenzhen 518132, China

^3^Guangdong Provincial Key Laboratory of Cell and Gene Therapy, Faculty of Pharmaceutical Sciences, Shenzhen University of Advanced Technology, Shenzhen 518107, China

^4^College of Medical Technology and Engineering, Henan University of Science and Technology, Luoyang 471023, China

^5^School of Pharmacy, Macau University of Science and Technology, Taipa, Macao SAR, China

^6^Science and Technology Innovation Center, Guangzhou University of Chinese Medicine, Guangzhou 510405, China

^7^Emergency Department, First Affiliated Hospital of Kunming Medical University, Kunming 650032, China

^＃^These authors contributed equally to this work.

*Corresponding authors: Jian-Lin Wu, Email: jlwu@must.edu.mo, Tel.: +853 8897 2406, Fax: +853 2882 5886; Na Li, Email: nli@must.edu.mo, Tel.: +853 8897 2405, Fax: +853 2882 5886.

**1. SIN metabolites identification**

11 metabolites were detected from rat serum compared with the sample collected at 0 min (**Table 1** and **Fig. S2**). These metabolites could be grouped as hydrogenated metabolites, oxygenated metabolites, demethylated metabolites and oxygenated-demethylated metabolites, and their structures were characterized by their chromatographic and MS/MS spectral behaviors in comparison with that of SIN as well as references [1]. These metabolites were identified as follows:

**Metabolite M1.** The two isomers M1a and M1b were eluted at 3.9 and 5.4 min, respectively. The isomers generated a [M+H]^+^ ion at *m/z* 332.1856. The molecular formula of C_19_H_25_NO_4_ was obtained on the basis of its accurate mass, indicating that hydrogenation tentatively occurred in double bond C7-C8 of the parent drug or carbonyl group in C6. The fragment ions at *m/z* 257.1081, 225.0823, 197.0884 and 183.0717 indicated the addition of 2H to SIN by comparing the corresponding fragments of 255.1016, 223.0754, 195.0804 and 181.0648 in SIN MS/MS spectrum. Comparing with their retention time, M1a was tentatively identified as 2H added to the carbonyl group in C6 of SIN while M1b was supposed to be 2H added to the double bond in C7-C8 of SIN. The chemical structure of M1 is shown in **Fig. S3**.

**Metabolite M2.** The isomers M2a, M2b, M2c, M2d and M2e were eluted at 2.1, 2.5, 4.5, 4.7 and 5.9 min, respectively. These isomers generated a [M+H]^+^ at *m/z* 346.1649 and shared the same molecular formula of C_18_H_23_NO_5_, indicating that one oxygen was added to the parent drug. The most amount metabolite M2e given the same ions at 255.0657, 239.0707 and 211.0741 with SIN. Moreover, the ion at *m/z* 330.1671 ([M+H-O]^+^)suggested that M2e could be sinomenine N-oxide, which was further confirmed by its RT and the reference [2]. M2c and M2d gave a high abundance of ion at 328.1556 ([M+H-H_2_O]^+^), indicating that the oxygen was added to the aliphatic chain rather than the benzene ring or *N* atom, for aliphatic hydroxylation metabolites showed a predominant loss of water while phenolic hydroxylation or *N*-Oxides exhibited small amount or no water loss [2]. In the same way, M2a, M2b isomers were temporarily identified as oxygenated SIN with the oxygen added the benzene ring by the present ion at *m/z* 289.1098 ([M+H-CH_2_CHNHCH_3_]^+^) and the absent ion at 328.1556 ([M+H-H_2_O]^+^) [2].

**Metabolite M3.** The two isomers M3a and M3b were eluted at 1.9 and 5.3 min, respectively. The molecular formula of these isomers were C_18_H_21_NO_4_, and their protonated molecule were [M+H]^+^ at *m/z* 316.1543, which indicating that one methyl group was removed from the parent drug. This was confirmed by their mass fragment ions in **Fig. S3**.

**Metabolite M4.** The two metabolites M4a and M4b were eluted at 1.2 and 4.9 min, respectively. They shared the same molecular formula C_18_H_21_NO_5_ and the protonated molecule [M+H]^+^ at *m/z* 332.1492. M4b exhibited the fragmentation ion at *m/z* 58.0651 indicating the piperidine ring is unchanged from the parent drug. The generated ion at *m/z* 316.1543 indicated the loss of an O group, and the following ions at *m/z* 259.0970, 241.0860, 231.1031, 225.0541, 197.0594, 189.0908, 181.0652 and 137.0585 were similar with that of M3b. By comparing with the reference[3], M4b was tentatively identified as N-Oxy-7-O-demethylsinomenine with its characteristic ions at m/z 166.0859 and 136.0614 in **Fig. S3**.

All the MS2 of metabolites shared the same mass spectrometric pattern with the parent sinomenine, indicating the structure of the metabolites was not changed.


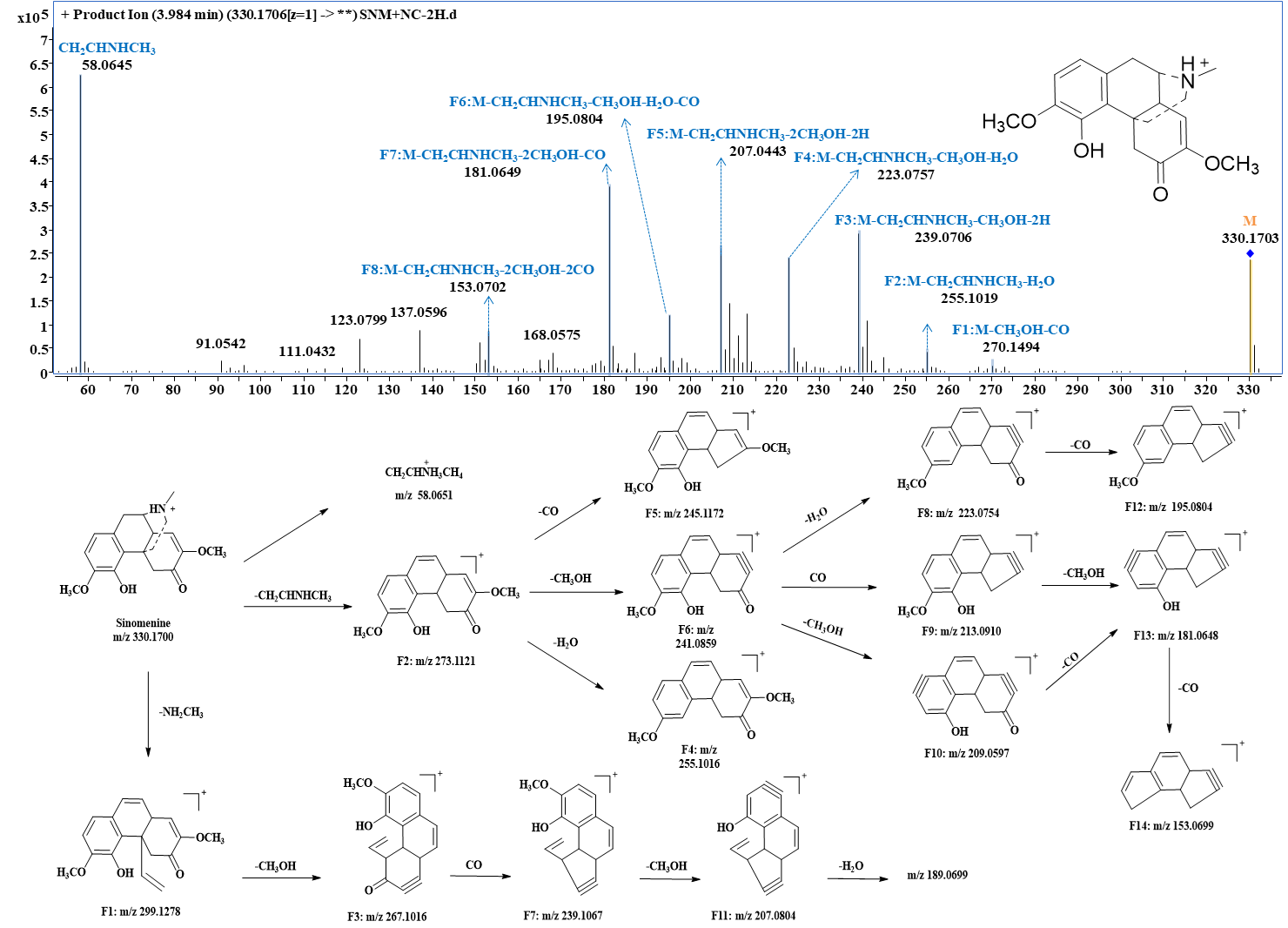


**Fig.S1.** Proposed MS/MS fragmentation pathways of SIN.


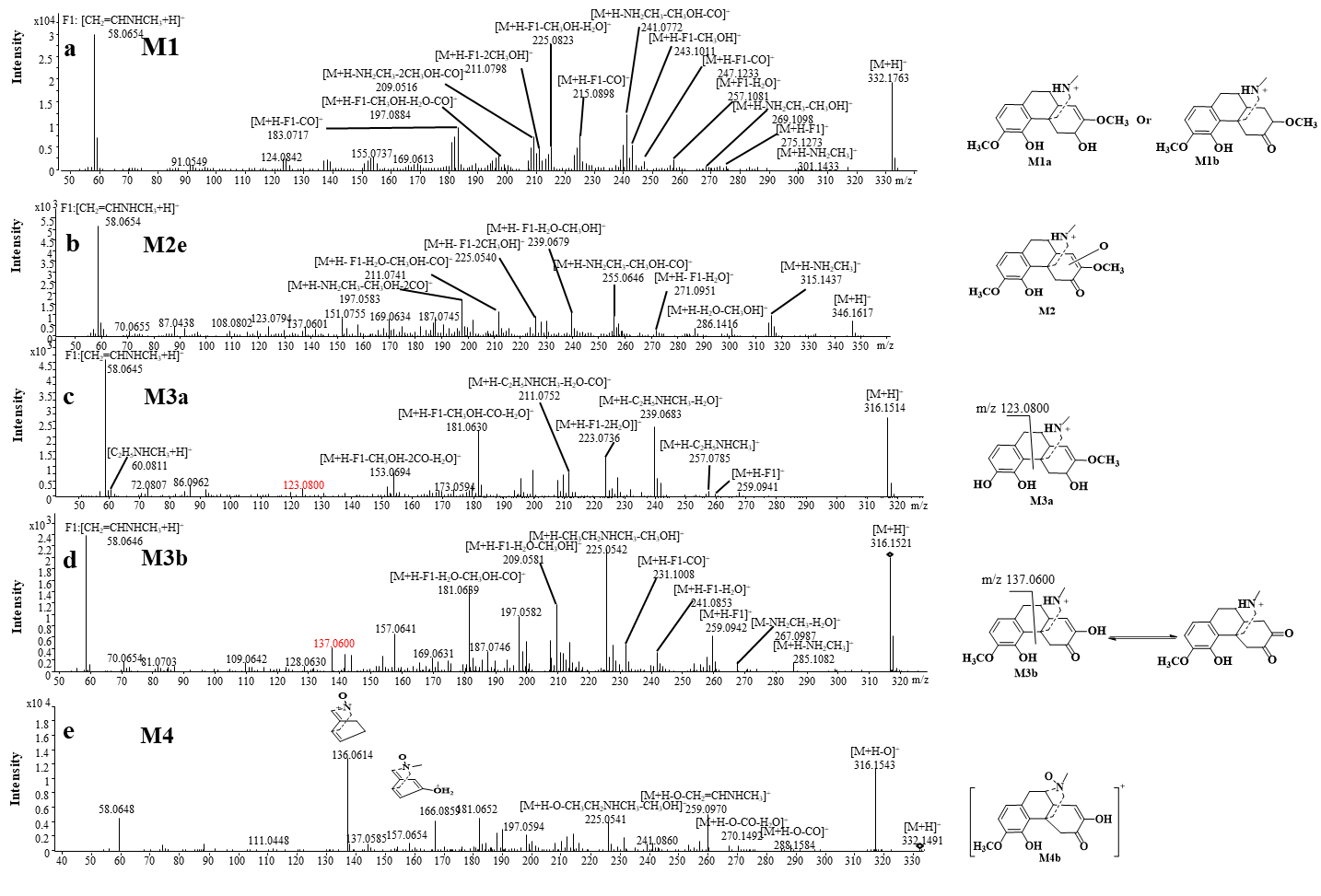


**Fig. S2.** MS/MS spectra of SIN metabolites: a hydrogenated metabolites (M1), b oxygenated metabolites (M2), c-d demethylated metabolites (M3) and oxygenated-demethylated metabolites (M4).


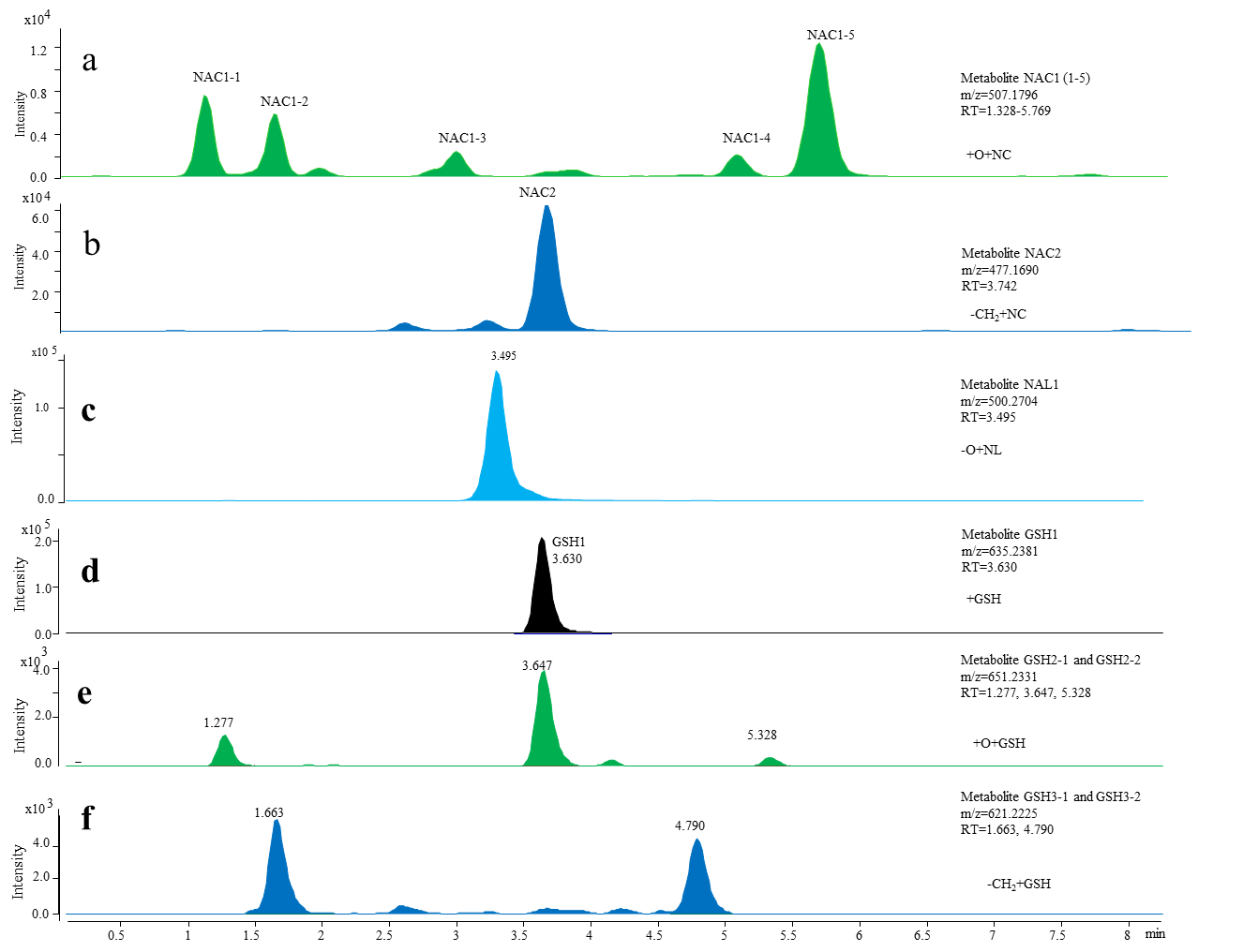


**Fig.S3.** Extracted ion chromatogram (EICs) NAC-hydroxylated SIN conjugates (a), NAC-demethylated SIN conjugate (b), NAL-SIN conjugate (c), GSH-SIN conjugate (d), GSH-oxygenated SIN conjugates (e) and GSH-demethylated SIN conjugates (f).


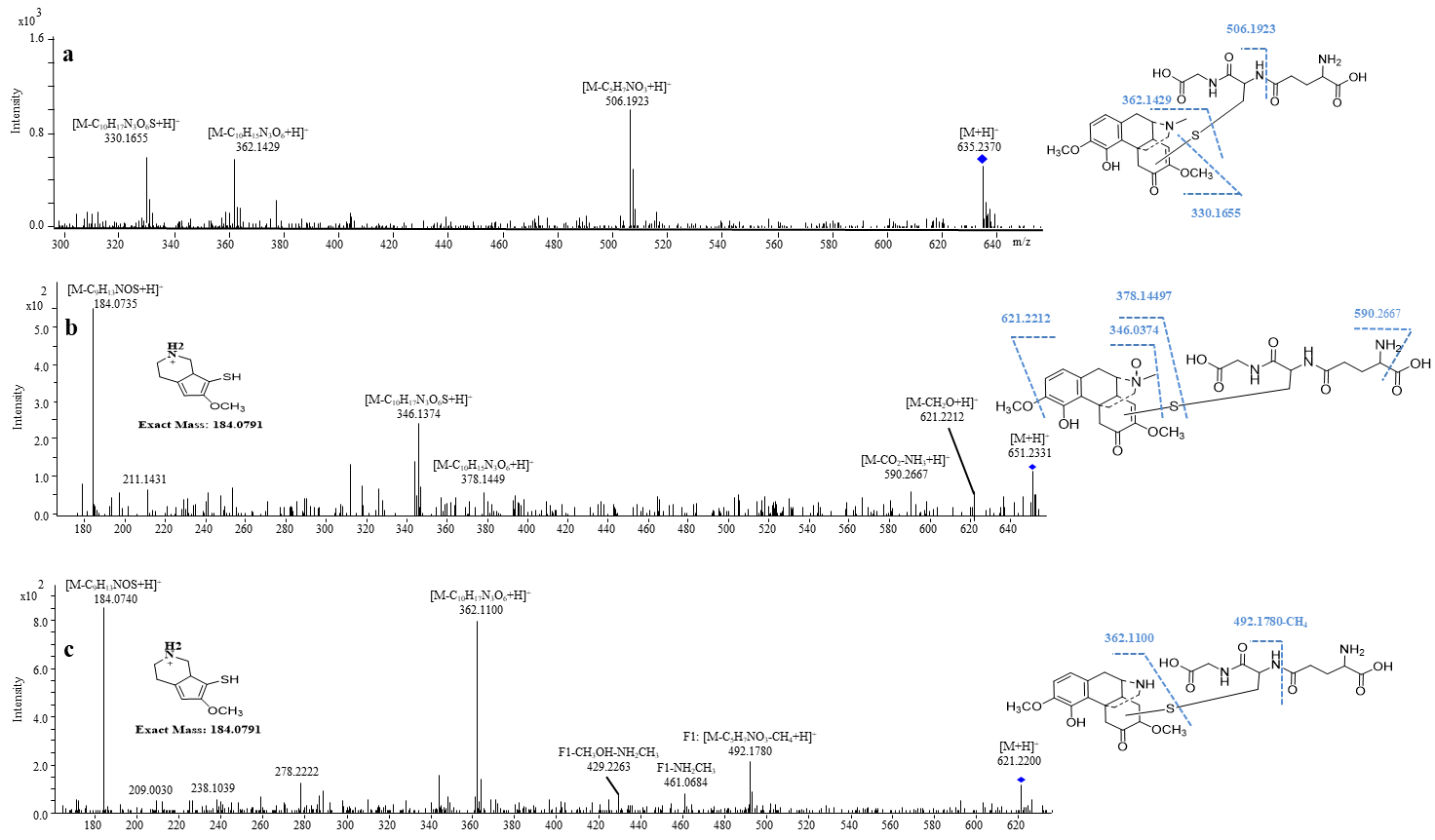


**Fig. S4.** MS/MS spectra of GSH-SIN reactive metabolites: GSH-SIN conjugate (a), GSH-oxygenated SIN conjugates (b) and GSH-demethylated SIN conjugates (c).


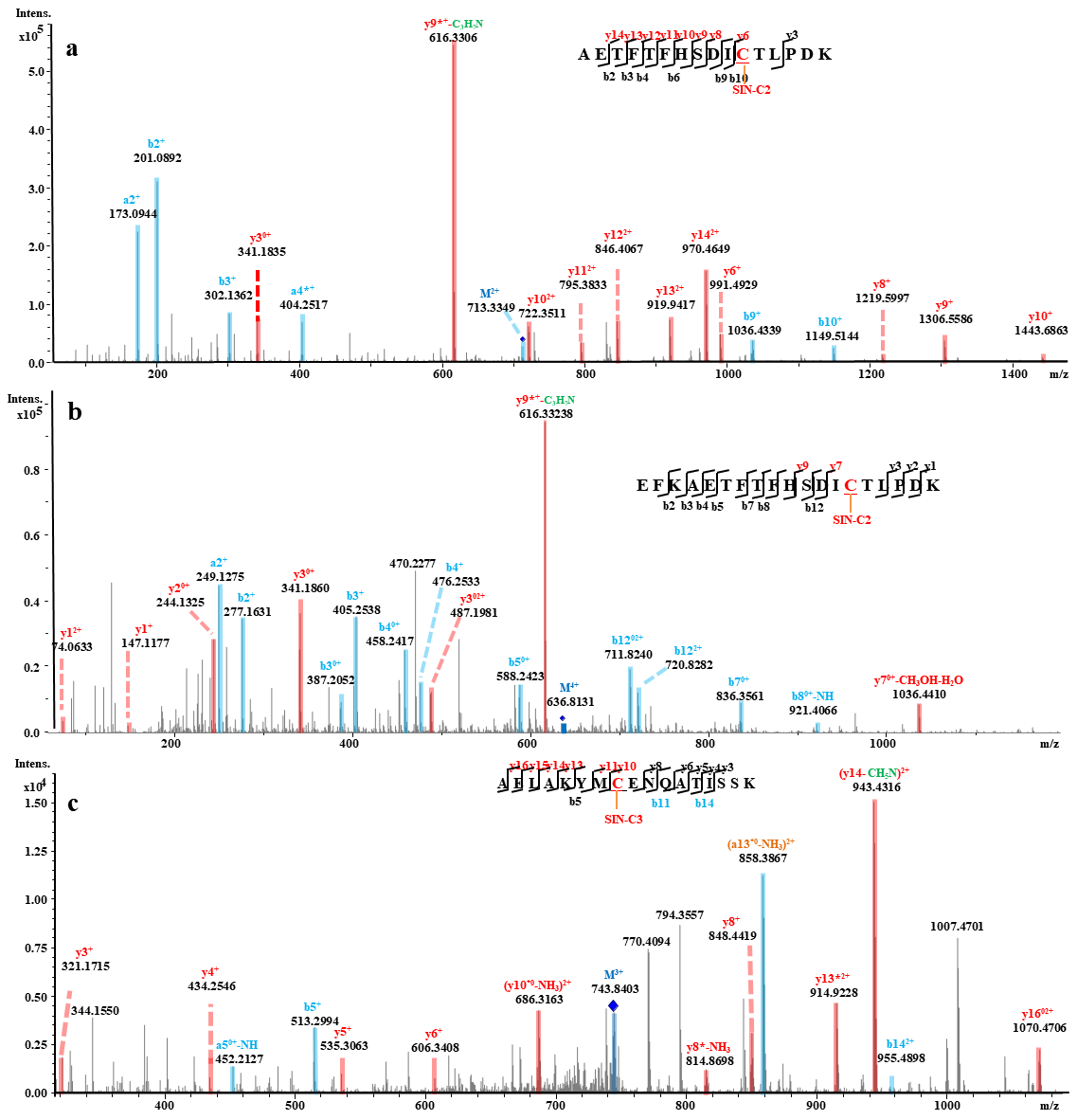


**Fig. S5.** MS/MS spectra for SIN RM-modified peptide of AETFTFHSDICTLPDK (a), EFKAETFTFHSDICTLPDK (b) and AELAKYMCENQATISSK (c) in RSA. SIN-related fragment ions are highlighted in green.


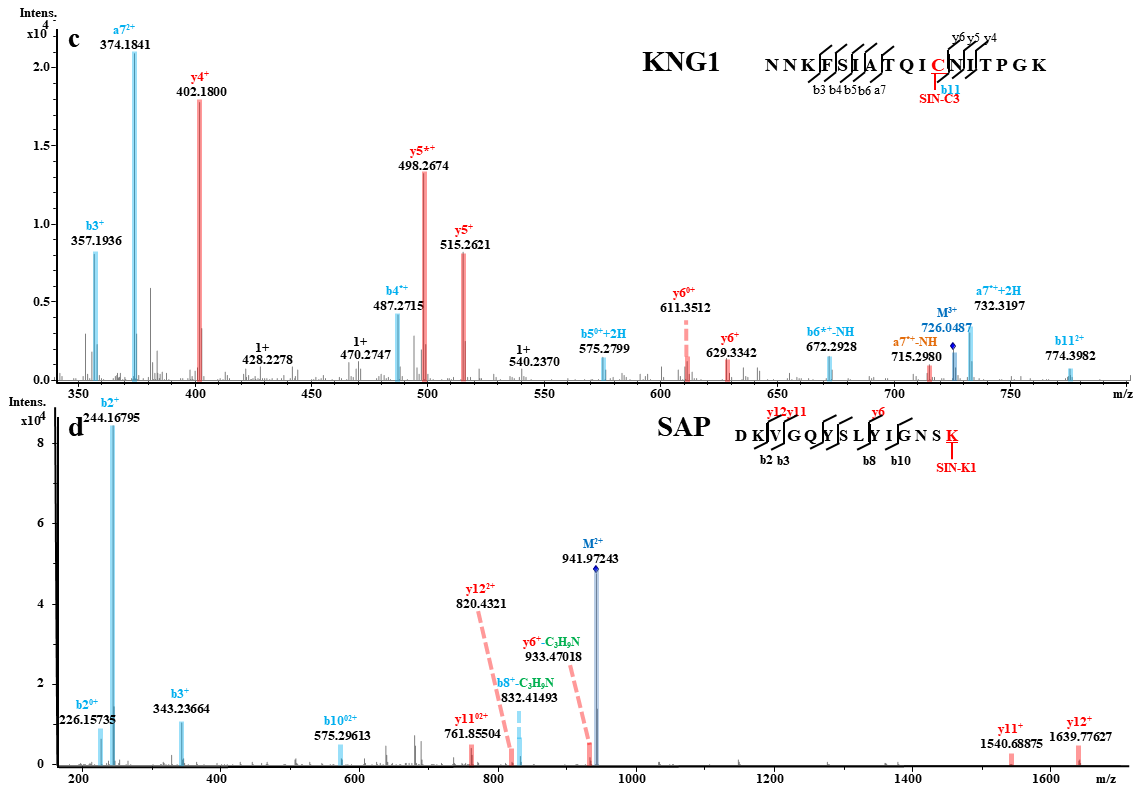

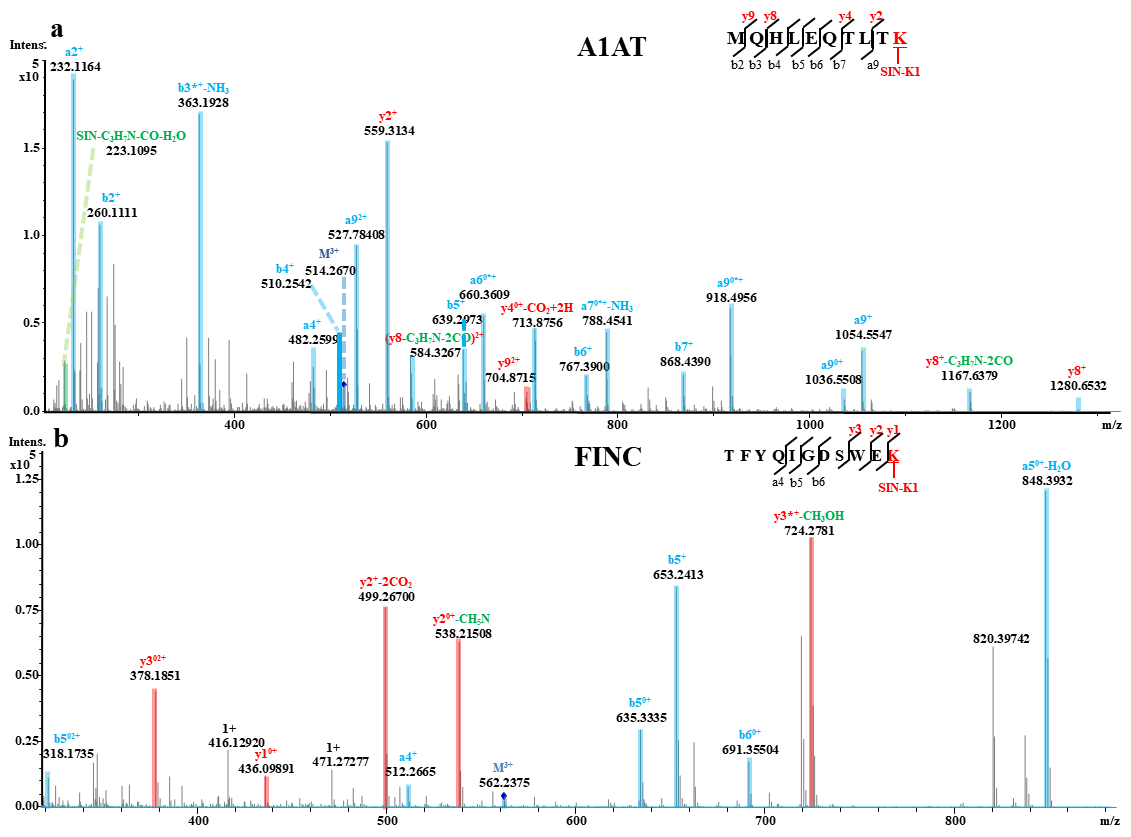


Fig. S6. MS/MS spectra of SIN RM-adducted peptides for protein A1AT(a), FINC(b), KNG1(c) and KNG1 (d) in rat serum. SIN-related fragment ions are highlighted in green.


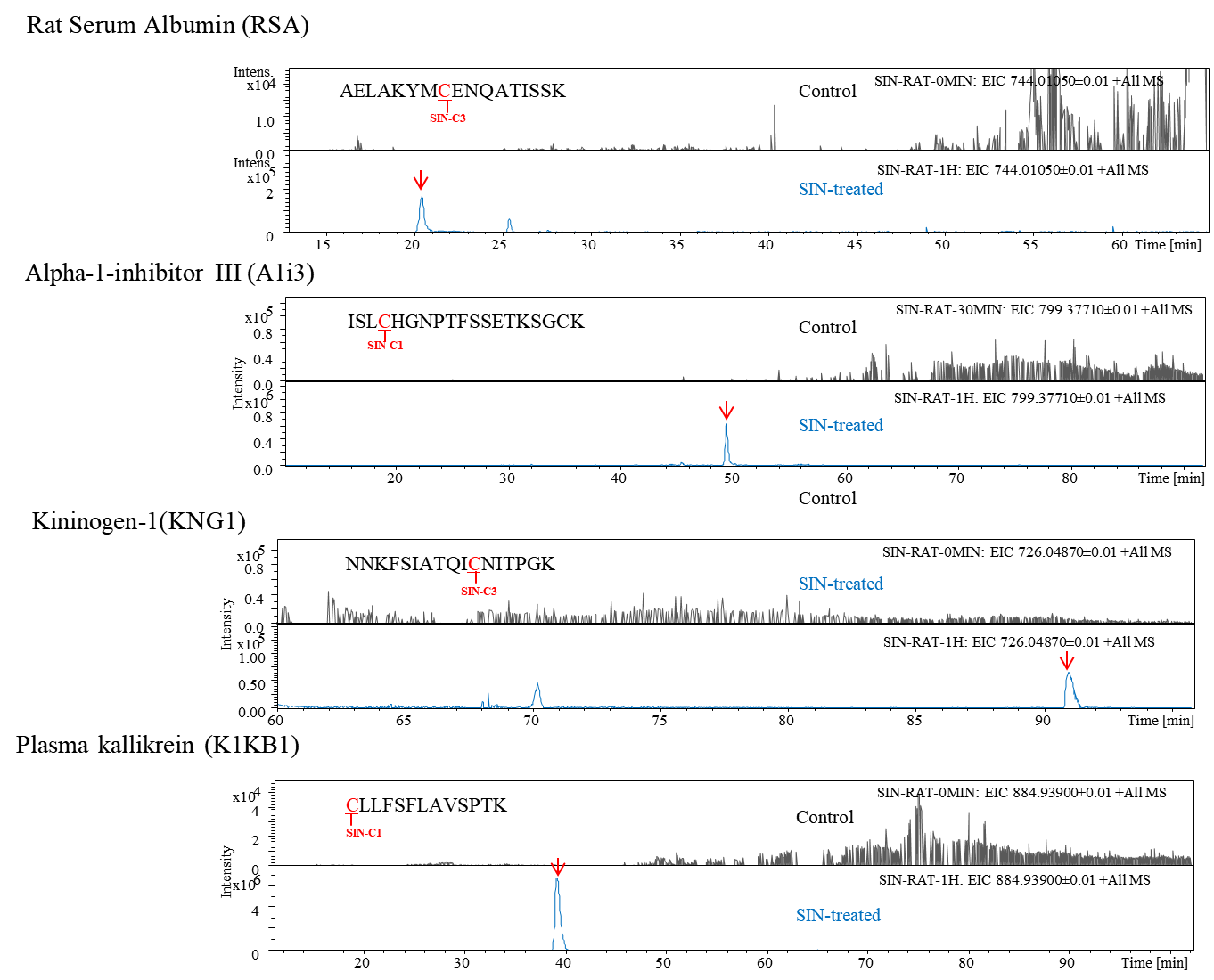


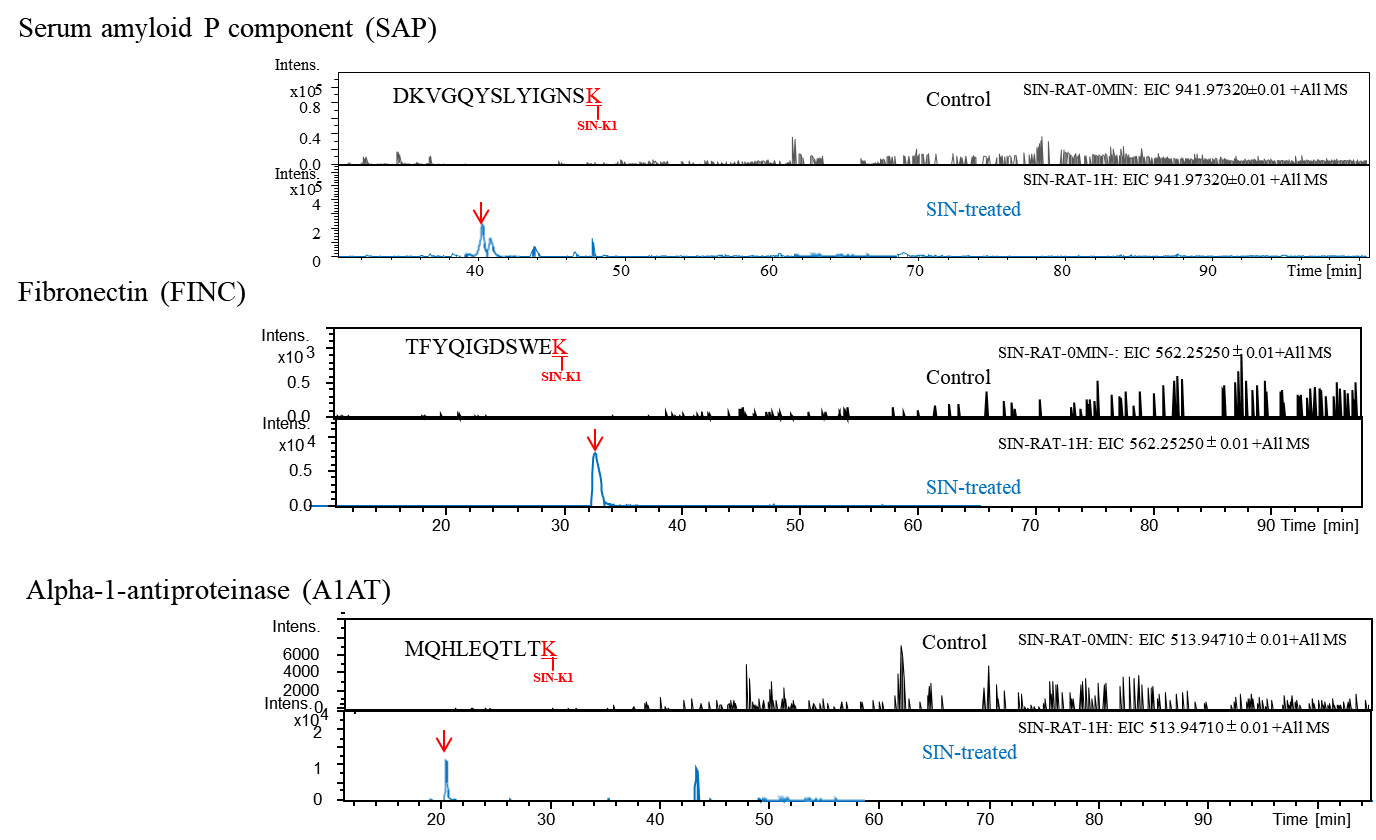


Fig. S7. Relative comparison of representative SIN-modified peptides between SIN-treated and control samples by extracted ion chromatograms (EICs). For each identified modified peptide, the EIC signal in the SIN-treated sample (blue) is compared with that in the corresponding control sample (black). SIN-modified peptides show clear or preferential signals in treated samples, whereas little to no corresponding signal is observed in controls. Red labels indicate the modified residue. These comparisons provide relative evidence that the identified adducted peptides are treatment-associated rather than background features.

**References**

1.Cheng WM, Qiu F, Yao XS: **Three major urinary metabolites of sinomenine in rats**. *Journal of Asian natural products research* 2007, **9**(1):13–18.

2.Ramanathan R, Su AD, Alvarez N, Blumenkrantz N, Chowdhury SK, Alton K, Patrick J: **Liquid chromatography/mass spectrometry methods for distinguishing N-oxides from hydroxylated compounds**. *Analytical chemistry* 2000, **72**(6):1352–1359.

3.Cheng WM, Qiu F, Yao XS: **Three major urinary metabolites of sinomenine in rats**. *Journal of Asian natural products research* 2007, **9**(1):13–18.
